# Doing well-being: Self-reported activities are related to subjective well-being

**DOI:** 10.1371/journal.pone.0270503

**Published:** 2022-06-24

**Authors:** August Håkan Nilsson, Erik Hellryd, Oscar Kjell

**Affiliations:** Department of Psychology, Lund University, Malmö, Sweden; Texas A&M University, UNITED STATES

## Abstract

Activities and Subjective Well-Being (SWB) have been shown to be intricately related to each other. However, no research to date has shown whether individuals understand how their everyday activities relate to their SWB. Furthermore, the assessment of activities has been limited to *predefined* types of activities and/or closed-ended questions. In two studies, we examine the relationship between self-reported everyday activities and SWB, while allowing individuals to express their activities freely by allowing open-ended responses that were then analyzed with state-of-the-art (*transformers-based*) Natural Language Processing. In study 1 (*N* = 284), self-reports of Yesterday’s Activities did *not* significantly relate to SWB, whereas activities reported as having the most impact on SWB in the past four weeks had small but significant correlations to most of the SWB scales (*r* = .14 –.23, *p* < .05). In Study 2 (N = 295), individuals showed strong agreement with each other about activities that they considered to increase or decrease SWB (AUC = .995). Words describing activities that increased SWB related to physically and cognitively active activities and social activities (“football”, “meditation”, “friends”), whereas words describing activities that decreased SWB were mainly activity features related to imbalance (“too”, “much”, “enough”). Individuals reported both activities *and* descriptive words that reflect their SWB, where the activity words had generally small but significant correlations to SWB (*r* =. 17 –.33, *p* < .05) and the descriptive words had generally strong correlations to SWB (*r* = .39–63, *p* < .001). We call this correlational gap the *well-being/activity description gap* and discuss possible explanations for the phenomenon.

## Introduction

Whether we work, make love, or procrastinate, our lives are filled with everyday activities. But how do our everyday activities relate to our Subjective Well-Being (SWB), which is seemingly the most important life goal according to both research [1, 2] and philosophers [3]? The relationship between the two may at first glance be a rather intuitive one. Activities can make us feel good, happy, excited, or euphoric. They can also make us feel bad, irritated, gloomy, or outright miserable. However, our intuition and beliefs sometimes blind us to our feelings [4], a phenomenon in theory also applies to activities we believe make us happy [5]. Research regarding which activities relate directly to SWB is relatively coherent [6–8]. But the question remains about whether individuals understand how their everyday activities impact and reflect their overall SWB.

Studies have shown that certain types of activities (like social activities such as spending time with others, especially family and relations [9, 10] and physical activities such as sex [8] and sports [6]) relate to having high SWB scores. Furthermore, the *activity features* matter. Lyubomirsky and Layous [11] argue in their positive activity model that, among other features, *dosage* and *variety* of activities associated with individuals with a high SWB affect the strength of those activities’ effects on SWB (see also [12]). Whereas most current research attempts to assess the relationship between *actual* activities and SWB, here, our aim is to examine individuals’ own subjective understandings of the relationship between everyday activities (whether performed or not) and SWB.

Moreover, the majority of these previous studies have explored the relationship between SWB and activities predefined by the researchers, such as social activities or activities believed to cause SWB [9, 13]. Here, we focus more broadly on everyday activities in relation to SWB, which includes assessing overall activities that are *not* predefined. To achieve this goal, we let individuals give free-form responses and then analyze the responses with modern Natural Language Processing (NLP) techniques. This technique allows us to quantitatively analyze free and open answers without imposing any activity category. The aim of the presented research is to i) examine the relationship between self-reported *everyday activities* and SWB, ii) examine whether individuals can describe how their everyday activities relate to their SWB, and iii) examine which everyday activities individuals relate to SWB.

### Well-being as activities

The question of what constitutes happiness, well-being, and a good life is hardly a modern one, and philosophers have given the matter substantial consideration throughout human history. According to Aristotle in the Nicomachean Ethics [3], for example, happiness, or *eudaimonia*, defined as living in accordance with oneself and one’s virtues, is the only thing humans truly aspire to; all other aspirations are simply byways of achieving happiness. Of interest for this study, Aristotle viewed eudaimonia as a continuous activity, one of living well and virtuously. Thus, for Aristotle, happiness is something that you do (cf. activities), rather than something you possess. Modern research has developed this notion and demonstrated a link between activities and self-expression, and between activities and how we experience joy [14].

#### Activities

Research on activities and SWB often lack a clear and elaborate definition of what constitutes an activity [8, 12, 15, 16]. Despite this, in general, researchers regard activities as what people spend their time doing [12] and how people behave [8]. We define activity as anything a person *does* or has *done* during a given time span. Since we focus on examining everyday activities, it is important to have a broad definition. The subsequent broad instruction to the participants allows them to define the concept rather than having us impose a definition on them (which is similar to how SWB is conceptualized within the SWB approach).

#### Subjective Well-Being (SWB)

In modern psychology research, well-being and happiness are often referred to and described as SWB. Despite claims that SWB taps into hedonism and lacks some of Aristotle’s eudaimonia [17], SWB is the most reliable and researched well-being construct [18]. SWB can be briefly defined as “the level of well-being people experience according to their subjective evaluations of their lives” [19, p. 391]. SWB concerns people’s own view of their well-being and a global evaluation of people’s lives.

SWB consists of a cognitive component and an affective component [20, 21] The cognitive component (how one thinks about one’s life) is often defined as *life satisfaction* [20] and can be described as “a global assessment of a person’s quality of life according to his [her, or their] own chosen criteria” [20, p. 543]. However, researchers have criticized SWB and Satisfaction with Life for being too hedonistic, focusing on achievement and self-actualization [14, 17, 22]. In contrast, when laypeople around the globe define happiness, they emphasize harmony twice as often as they do satisfaction [22, 23]. In response to observations like these, the construct *Harmony in Life* [23], focusing on inner harmony, balance, and social relationships, has been demonstrated to complement Satisfaction with Life [24].

The affective component of SWB, defined as experienced emotions, comprises *Positive Affect* and a lack of *Negative Affect* [25]. Positive Affect and Negative Affect do not represent two opposites of the same spectrum, but rather distinct dimensions only weakly correlated with each other. The preponderance of Positive Affect is considered to constitute high SWB [20, 26].

### Characteristics and features of activities in relation to SWB

There is a well-established association between social activities and SWB. For instance, recently a large meta-study consisting of 556 effect sizes found that social activities associate positively with SWB [9]. One notable finding is that among the top 10% of happiest people, not even one lacked a close, good-quality relationship in their lives [10]. The very same happy people spent the least time alone, the most time socializing, and were rated by others as better at social relationships than the other groups. Furthermore, the broaden-and-build theory of positive emotions holds that Positive Affect enhances one’s thought-action repertoires, which include building and maintaining social relationships [27]. Individuals inducted into a state of positive emotions can think of more activities related to playing and social activities than can individuals induced into a neutral or negative affective state; furthermore, those induced into a state of negative affect conversely wanted to be antisocial [28]. The most unhappy people spent the most time alone [10].

#### Active versus passive activities

High SWB associates with many cognitively and/or physically *active activities*. Cognitively active means that individuals generally are actively engaged and focused on the activities they do without necessarily being physically active. For example, a recent meta-analysis [7] showed that mindfulness (e.g., in the forms of mind-body scans and mindful imagery) had the strongest effect size among SWB interventions. It has also been shown that counting one’s blessings can improve SWB [11], and the categories meditation and listening to music/podcasts are rated among the highest in momentary SWB [8]. Among the active activities, “Sex” was the activity rated highest on momentary SWB for various SWB dimensions, followed by “partying” [8]. Another recent meta-analysis showed that doing sports-related to SWB [6], and individuals induced into a state of positive affect showed more urges to be outdoors in nature and play sports or do exercise [28]. Conversely, cognitively and physically *passive* activities appear to relate to low SWB. For example, individuals induced into a state of negative affect wanted to neither eat, work, nor do activities in general [28]. Grimm et al. [8] found that the second-lowest rated activity regarding SWB was “[being on] Facebook”, second only to “[being] sick”. Other low-rated activities included “Commuting” and non-specific activities related to “Internet”.

#### Activity features and SWB

The *positive-activity model* posits that the effect on SWB of activities considered to enhance SWB depends on various activity and personal features, such as activity variation, activity dosage, and social support [11]. It should be noted that the relationship between dosage and activity level is not necessarily positive. For example, an article reviewing eight studies about variety in activity showed that variation over a longer time span, such as a day, increases happiness, whereas variation in activities over shorter time spans, such as an hour, decreases happiness [12]. Similarly, another study [13] showed that counting one’s blessings once a week increases SWB more than counting them three times a week, directly illustrating how the variation-related feature *dosage* affects the relation between activities and SWB.

#### Everyday activities and SWB

Momentary SWB relates to the currently-experienced activity. Using an *experience-sampling method*, participants reported their current activity and rated their momentary SWB on various SWB dimensions [8]. “Sex/making love” scored the highest on all dimensions. Typical pleasure activities included “drinking alcohol/partying” and “listening to music/podcasts”, whereas typical meaning activities included “meditating/religious activities” and “care-giving/volunteering”, and typical engagement activities included “gardening/outdoor housework” and “hobbies/arts/crafts”. Ordinary everyday activities in the form of duties that most people do, such as “housework/chores”, “studying” and “paid work” were rated among the lowest contributors to momentary SWB. A methodologically similar study found that among teenagers the categories of social and active activities correlated with high SWB and with being in a state of flow, whereas studying correlated with low momentary SWB; however, more study time over longer time periods resulted in higher overall SWB [29]. This study illustrates a case of discrepancy in the SWB–activity relationship, demonstrating that the relationship depends on the time frame of the activity and SWB. Current activities can affect SWB in the long term, so even if an activity is not associated with momentary SWB, it might be associated with overall (or longer-term) SWB, and vice versa. However, research has generally shown agreement on how everyday activities relate to SWB.

### Can individuals describe the activity/well-being relationship?

By using modern NLP techniques, it is possible to predict individuals’ overall SWB from their self-reported (past) everyday activities. One shortcoming of previous studies is that individuals have not been allowed to describe their overall everyday activities of a given time span retrospectively and in an open-ended manner. Previous studies have asked participants if they have done certain types of activities [9, 15], what they would like to do [28], or about their everyday activities at random moments during the day for a week [8, 29]. Prior research has tackled the activity/well-being relationship from different angles, and it seems that activities are indeed related to SWB [6–11, 29]. However, to our knowledge, no one has tested whether individuals can describe the relationship between their past everyday activities and their overall SWB.

In these investigations, we first test a broad assessment of retrospective everyday activities by letting individuals report all the activities they did yesterday (Study 1). Second, we test an assessment of individuals’ descriptions of the activity/well-being relationship by asking them to report which activities they have done that affect (Study 1) or reflect (Study 2) their well-being. Both studies examine how individuals recall activities that relate to their self-reported subjective well-being (i.e., their subjective understanding). These two latter questions can help us understand to what extent individuals understand the relationship between their everyday activities and their SWB.

### Measuring and describing psychological aspects from text responses

When asking somebody which activity(ies) they do, or normally do, it is more natural and more ecologically valid to ask questions with an open-ended response format rather than a closed-end predefined response format. Natural Language Processing (NLP) allows researchers to conduct quantitative analyses of such answers without having to (rather arbitrarily) code or categorize the data. Analyzing text answers in response to questions about various psychological constructs using NLP has yielded good psychometric properties with similar or higher reliability and validity than the numerical-rating-scale counterpart (see [30]).

*Computational Language Assessments* assess a construct by language data rather than numerical data. Open-ended questions asking for free responses to assess psychological constructs have been validated by predicting corresponding numerical scale scores from the text responses (e.g., open-ended responses to questions such as “Overall in your life, are you in harmony or not?” can be used to predict the Harmony in Life scale scores with a correlation of Pearson *r* = .85 [31]). Moreover, measuring Harmony in Life with open-ended questions has been shown to be significantly correlated with theoretically relevant behaviors (*cooperation*), which is not true for the corresponding rating scale [32].

### The present studies

This article comprises two studies. Study 1 examines whether self-reported everyday activities (e.g., as captured in the words comprising Yesterday’s Overall Activities) relate to SWB, and how individuals understand the relationship between everyday activities and SWB by asking for the activities with *the most impact* on their SWB over the past four weeks. Study 2 further explores how individuals understand the relationship between their activities and SWB by asking for regular activities that *reflect* their SWB, which we correlate with numerical as well as computational language assessments of SWB. Study 2 also tests which regular activities individuals perceive to increase and decrease their SWB, and how well different individuals agree about these answers.

## Study 1

The hypotheses of Study 1 include:

### Overall everyday activities predict SWB

#### Hypothesis 1a

The activities individuals report they did yesterday predict their SWB.

#### Hypothesis 1b

The valence of the activities individuals report they did yesterday correlates positively with their SWB.

### Everyday activities that participants perceive to affect their SWB predict SWB

#### Hypothesis 2a

The activities individuals say affected their SWB the most during the past four weeks predict their SWB.

#### Hypothesis 2b

The valence of the activities individuals say affected their SWB the most during the past four weeks correlates positively with their SWB.

A pre-registered hypothesis regarding variation in answers about Yesterday’s Activities (including analysis) can be found in Table S1.1 in S1 File.

## Method

### Participants

Three hundred adults in the UK were recruited through *Prolific* [33], an online platform for recruiting research participants that also provides demographic data. Of the 300 initial participants, 289 completed the entire survey. In addition, five failed to correctly answer a control question (further described below) and were, as pre-registered, excluded from further analyses, thus leaving 284 participants. Participants were compensated £0.8 to participate in the study, which took on average 7.6 (*SD* = 3.9) minutes to complete. Of the 284 participants, 193 were female and 91 were male with a mean age of 35 (*SD* = 12, range 18–74) years. Regarding employment status, 118 worked full-time, 69 worked part-time, 30 were unemployed, and 67 had some other employment status, such as non-paid work. Forty-five participants were students. The average subjective socioeconomic status was 5.01 (*SD* = 1.65), where participants placed themselves on a ladder representing how people stand in society, from 1 (worst off) to 10 (best off).

### Instruments

To assess everyday activities, including activities that the participants had performed yesterday and activities with the most influence on SWB in the past four weeks, we adapted the open-ended *Twenty Statements Test* [28, 34] with inspiration from open-ended SWB questions [30].

#### Yesterday’s activities

Participants were asked to think about activities as anything they did yesterday, and were then asked, “Please list activities you did yesterday morning/during the day/evening.” In total, participants could write a maximum of 30 activities (10 for each part of the day). Participants reported on average 14.07 (*SD* = 6.16) activities.

#### Activities in the past four weeks

The question regarding activities that had had the most influence on their SWB in the past four weeks was “Please list the activities that have had the most impact on your well-being in the past four weeks”, followed by the activity definition and clarification that the effect of the activities on SWB could be both positive and negative. In total, participants could list a maximum of 20 activities. Participants reported on average 6.78 (*SD* = 3.95) activities. Two participants answered all questions except this question, leaving 282 participants for the analyses.

#### Subjective Well-Being (SWB) scales

The abbreviated three-item versions of the Harmony in Life Scale (HILS-3) and the Satisfaction with Life Scale (SWLS-3 [24]), derived from their five-item original versions [21, 23], were used. The abbreviated versions are validated to be presented together with shared instructions without compromising the psychometric properties. The scales include three items each (e.g., “I am in harmony” for HILS-3, “I am satisfied with my life” for SWLS-3) answered on Likert scales ranging from 1 to 7 (*strongly disagree* to *strongly agree*). Both Cronbach’s alpha and McDonald’s omega total were .93 for the HILS-3 and .90 for the SWLS-3.

#### The Positive and Negative Affect Schedule (PANAS)

In the PANAS [26], participants are asked to indicate to what extent they feel or have felt 20 different emotions on Likert scales ranging from 1 to 5 (*very slightly or not at all* to *extremely*). Of the 20 words related to various emotions in the PANAS, 10 form the *Positive Affect Scale* (henceforth “PA”; e.g., “interested”, “excited” and “proud”), and the remaining 10 form the *Negative Affect Scale* (henceforth “NA”; e.g., “distressed”, “guilty” and “upset”). In this study, participants were asked about experienced affect for the *past four weeks*, primarily to correspond with the time frame in the question regarding activities in the past four weeks. Cronbach’s alpha was .91 for both the PA scale and the NA scale, and McDonald’s omega total was .93 for both scales.

#### SWB composite

It is common to use an SWB composite measure by subtracting the standardized NA score from the sum of the standardized SWLS score and the PA score [35, 36]. We used a similar SWB composite score, but we substituted the SWLS with the average of the standardized scores from the SWLS-3 and the HILS-3 (Positive Affect score + ((SWLS-3 + HILS-3)/2)—Negative Affect score). This composite score was not pre-registered.

#### Control item

One control item was included in the survey: “Please answer the alternative ‘4: neither agree nor disagree’ below.” This item occurred among the HILS-3 and the SWLS-3 items with the 7-point Likert scale used for the rating scales. Participants who failed to answer correctly were removed from further analysis. This type of control item has previously been demonstrated to increase reliability and statistical power in the data set [37].

### Procedure

Participants were asked to partake in a study regarding their lives, SWB, and activities. After being informed about the study and providing their consent, participants answered the questions about activities they performed yesterday. Then, they answered the question about the activities that had most affected their SWB in the past four weeks, followed by the SWB scales, and lastly, they were debriefed. The HILS-3 and the SWLS-3 were displayed together in a randomized order. The order of the HILS-3/SWLS-3 and the PANAS was randomized. All data collection occurred between 4 pm and 7 pm, on December 4th, 2019.

#### Ethical statement

The study complied with Swedish laws and research ethics regulations. Ethical approval from the Swedish National Ethics Boards is not required in a study like this, since it did not include any collection of sensitive personal information, was not associated with risks of psychological or physical harm, nor was it intended to manipulate/influence participants. In the consent form, participants were first told about the study, given the researchers’ contact information, told about their right to withdraw from the study at any time without having to give any reasons, and told that no personally sensitive information would be collected. Subsequently, they were asked to provide their consent before participating in the study.

### Natural language processing

NLP was used to analyze the word responses in both Study 1 and 2. All analyses were made in R [38] using RStudio [39], and the NLP-related analyses were made using the R-package *text* [40]. The functions of *Text* are optimized for social scientists and human-level analyses. The *text* package’s workflow first involves transforming text data to *word embeddings* (numeric representation of the meanings of words, as further explained below) using state-of-the-art pre-trained language models. These word embeddings are then used in downstream statistical tasks, such as predicting numerical rating scales, comparing different texts, testing whether specific words are associated with different groups and whether that difference is significant, and plotting associations between those words and groups [40]. Since NLP analyses are not widely used outside computer sciences, a brief description of the analytical methods used in this study follows (a detailed description can be found in [40]).

#### Word embeddings

A word embedding is a numerical representation of a word or a group of words. As such, word embeddings represent words through (many) numerical values, with the aim of capturing the latent meaning of the word(s) or text(s). The word embeddings are vectors in which the numbers can be seen as coordinates in different dimensions that describe the position of a word in a multi-dimensional space; the closer two different word embeddings are positioned in this space, the more similar they are in meaning. The word embeddings of individual words can be aggregated to represent several words (for more details on word embeddings, see [40]). These word embeddings are the basis for the language-based statistical analyses in this study; for example, they are used to predict SWB scales score.

To transform the text responses to word embeddings with high quality (i.e., representing meaningful information about the word[s]), the language model needs to be trained on large amounts of data. In this study, Google’s pre-trained language model called *Bidirectional Encoder Representations from Transformers* (henceforth “BERT”; [41]) is used. The pre-training of BERT was based on the English Wikipedia corpus comprising 2500M words and the BooksCorpus [41], a corpus-based on 11038 books comprising 1000M words [42]. BERT’s major advantage over most previous language models is that it *contextualizes* word embeddings such that, for example, it gives the word *play* a different word embedding in the phrase *watch a play* than in the phrase *play soccer* (for more details, see [41, 43]). Compared to previous models, BERT yields substantial improvements in performance on many language tasks [41], making it a suitable model for multiple purposes.

BERT represents each token (c.f. word) with 12 layers (where each layer represents a slightly different semantic value depending on the context), each of which comprises 768 dimensions. In this study, layers 11 and 12 were used (which is recommended and have performed well in human-level tasks [40]) by concatenation. To represent several words such as an entire text response, these word embeddings were aggregated by taking the mean value of each dimension.

#### Semantic similarity

Semantic Similarity Scores (SSS) capture how closely two different word embeddings are positioned in the word embedding space, and thus how similar they are in meaning. “How closely” is here measured as the cosine of the angle between the two word embeddings, and a higher SSS indicates a higher similarity in meaning.

To examine the degree to which different word responses relate to specific psychological constructs, SSS between the word responses and word norms that describe psychological constructs can be used. For example, the Harmony and the Disharmony word norms comprise over a thousand words each that have been generated by participants who described these constructs in previous studies [31]. The higher SSS a participant’s description of their personal harmony has with the Harmony word norm, the higher Harmony in Life they are considered to have [31]. This type of SSS is called Unipolar SSS. It is also possible to compute Bipolar SSS, which comprises the unipolar SSS for Harmony minus the SSS to the opposite word norm (in this example, to the Disharmony word norm).

#### Language-based predictions

To examine the relationship between text responses and numerical variables, the word embeddings (e.g., of the activities) can be trained to predict a numerical scale (e.g., the SWB scales) using ridge regression [44]. The dimensions of the word embeddings represent predictor variables. To reduce overfitting and to evaluate the validity of the model, 10-fold cross-validation is used, where the data is randomly split into train, development, and test sets. Models with different hyperparameters are trained and evaluated on the train and development sets, and then finally tested on the test set (on data that was not used during the development of the models; for a detailed description, see [40]). The predicted scores from the test sets are then used in a bivariate one-tailed Pearson correlation with the observed numerical scale scores. Note that since the correlations are based on predicted values, significant correlations should always be positive.

Logistic (ridge) regression is used to train word embeddings to classify binary criterion variables. The final evaluation is based on Area Under the ROC curve (AUC) and balanced accuracy.

Models trained on other data sets can be used to predict certain features (e.g., valence or arousal) of another set of text. For this study, we trained a model to predict the valence from word embeddings using the *Affective Norms for English Words* [ANEW; 45]. ANEW includes over 1000 words for which participants rated the valence using a 9-point scale. The word embeddings of the ANEW words were trained to predict the participants’ rated valence scores (as previously described). The valence predictions from the final model yielded a very strong correlation to the participant-rated valence scores (*r* = .83, *N*_words_ = 1,029, *p* < .001), which supports the reliability of the valence model. In these studies, we apply this valence model to estimate the valence of the participants’ answers, which then is correlated with their SWB scores.

#### Word plots

Word plots visualize text data and make it easier to understand the content. A *Supervised Dimension Projection Plot* depicts words that are used significantly different when the entire word set is divided into two groups [40]. The groups can be either categorical or numerical (for example, a median split of a numerical scale) such that the entire set of words is divided into a high and a low scoring group. For example, one group may consist of text data pertaining to activities that increase SWB, and the other group’s words pertain to activities that decrease SWB. The word embeddings of these two groups are first aggregated separately and then subtracted to make up the aggregated direction embedding. This point in space is seen as a supervised dimension that is represented by a direction line from the point through the origin. Each word’s embedding is subsequently projected onto this line using the dot product. A permutation procedure is used to compute *p*-values for each word so that a randomly-permuted null distribution of supervised dimension projections is created. Each word’s dot product projection compares to the null distribution to find a *p*-value while correcting for multiple comparisons using Holm correction [46; for more details see, 40]. Note that these plots also can visualize non-significant words, for example, the most frequent non-significant words.

### Cutoff and effect size

As pre-registered, alpha was set to .05, and effect sizes of significant correlations were interpreted as .10 –.29 = small, .30 –.49 = medium, .50 and above = strong.

The following packages were used for the remaining analyses: *Hmisc* [47], *dplyr* [48], *tidyverse* [49], *car* [50], *magrittr* [51], *tibble* [52], *devtools* [53], *rio* [54], *stringi* [55] and *psych* [56].

## Results

All numerical scales used for the analyses meet the assumptions of normal distribution, having all skew and kurtosis scores within the range of −.80 and .91, and consequently analyzed using Pearson correlations. All SWB scales yield moderate to strong correlations to one another (Table 1), the strongest being between the HILS-3 and SWLS-3 scales (*r* = .81, *p* < .001) and the weakest between the NA and PA scales (*r* = -.32, *p* < .001). All skew and kurtosis scores can be found in Table S1.2 in S1 File.

**Table 1:** Mean, SD, and Pearson correlations between SWB scales scores.

Variables	M	SD	1	2	3	4
**1. HILS-3**	12.32	4.26				
**2. SWLS-3**	12.17	4.48	.81			
**3. PA**	30.03	8.07	.53	.57		
**4. NA**	20.64	8.01	-.46	-.43	-.32	
**5. SWB**	0.00	2.35	.81	.81	.79	-.75

*Note*. *N* = 284. All *p* < .001 (2-tailed). HILS-3 = Harmony in Life Scale three item version, SWLS-3 = Satisfaction with Life Scale three item version, PA = Positive Affect, NA = Negative Affect.

### The relationship between self-reported activities and SWB scales

Neither Yesterday’s Activities (H1a) nor their predicted valences (H1b) significantly predict any of the SWB scales (all *p* > .05, Table 2), providing no support for H1. This means that the self-reported activities that people did yesterday are not significantly associated with SWB.

**Table 2:** Pearson correlations between language-based predictions of SWB and observed SWB scale scores.

Word responses	Method	SWB	HILS-3	SWLS-3	PA	NA
**Yesterday’s**	Training	.12	.07	.01	.02	-.01
**Activities**	Valence	.02	-.02	.00	-.01	-.07
**Activities in the**	Training	.12	-.09	.02	.14*	.09
**past four weeks**	Valence	.24**	.15*	.22**	.23**	-.15*

*Note*. N = 284

* *p* < .05

** *p* < .01. HILS-3 = Harmony in Life Scale three item version, SWLS-3 = Satisfaction with Life Scale three item version, PA = Positive Affect, NA = Negative Affect, SWB = Subjective Well-Being composite score, Valence = predicted valence of text responses. Analyses adjusted for multiple comparisons using Holm correction.

Activities that most affected SWB in the past four weeks (H2a) and their predicted valences (H2b) were hypothesized to predict SWB. The word embeddings of the activities themselves do not generally predict SWB except for a small, significant correlation to PA (*r* = .14, *p* < .05, one-tailed), giving generally no to very little support for H2a. The predicted valence of these activities’ word embeddings, however, correlate to all SWB scales (*r* = -.15–.24, *p* < .05, two-tailed), supporting H2b. Thus, participants that have high SWB scores describe their activities with the most effect on their SWB in the past four weeks with words that have a higher predicted valence score than participants with low SWB. Taken together, this result means that individuals’ descriptions of their everyday activities that they perceive to have had the greatest effect on their SWB in the past four weeks do indeed relate to their SWB, although only to a small degree.

### Yesterday’s activities and activities with the greatest influence on SWB in the past four weeks

**Fig 1:**
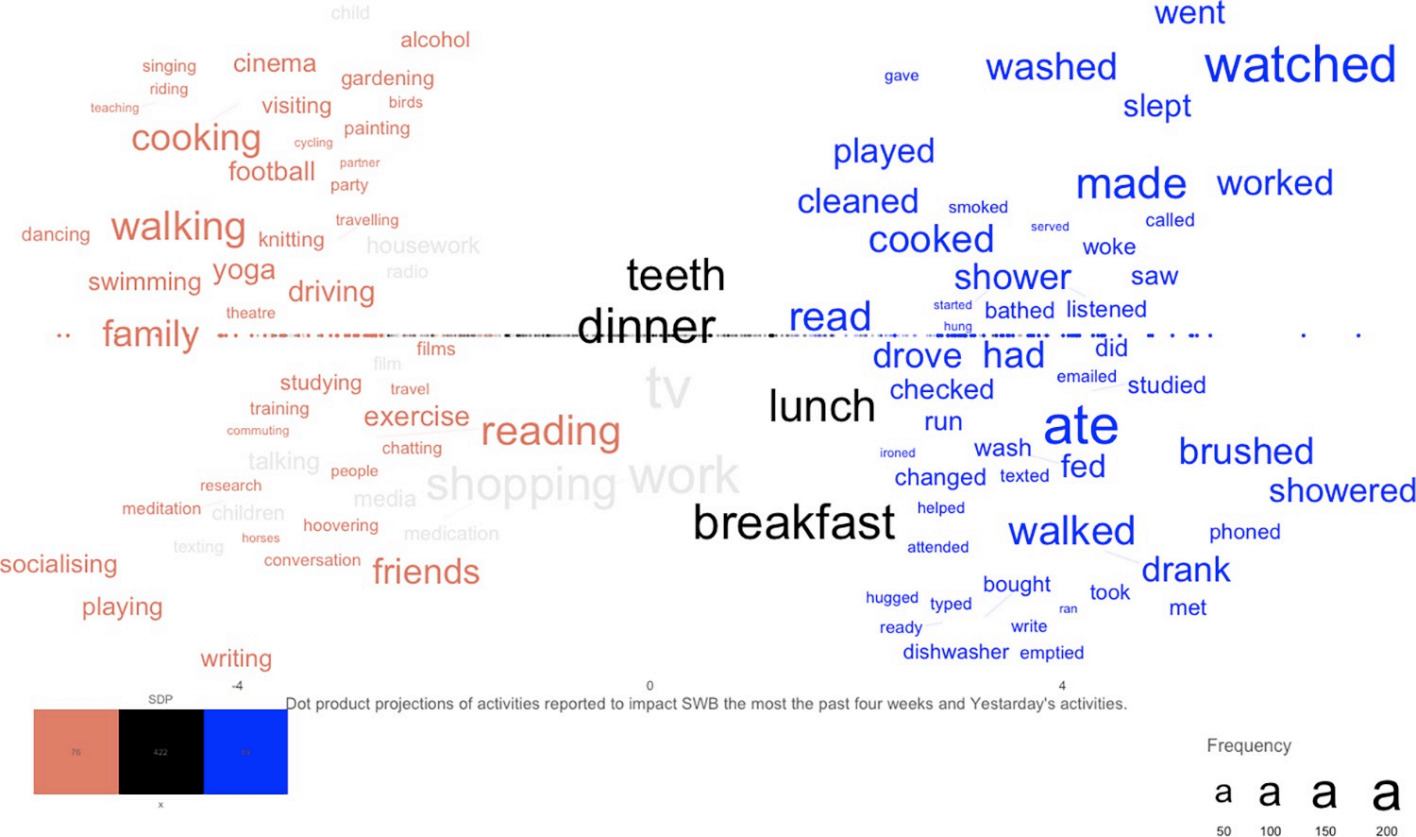
Activities reported to impact SWB the most the past four weeks and Yesterday’s Activities. *Note*: Supervised dimension projection plot for Yesterday’s Activities and activities that most affected the participants’ SWB in the past four weeks. The words appearing a minimum of 4 times in the questions, combined, have been significance tested against a permuted null distribution, *N*_*permutations*_ = 100,000. Words significantly belonging to Yesterday’s Activities are plotted on the right side in blue. Words significantly related to activities having the greatest effect on the participants’ SWB in the past four weeks are plotted on the left side in red. Black words in the middle are frequent words *not* significantly belonging to any of the groups. Grey words belong to one word category but more frequently occur in the other category (with significant statistical support). Word size represents frequency. The position on the dimension projection (the position on the x-axis) represents the dot product score. For better visualization, the words are separated on the y-axis, but the y-axis does not represent any information.

Yesterday’s Activities and activities with the greatest influence on SWB in the past four weeks are plotted in Fig 1 in a supervised dimension projection plot. The words significantly related to ‘Yesterday’s Activities’ (the blue words to the right) include many ordinary everyday activities related to home activities, e.g., “cleaned”, “slept”, “cooked”, “brushed (teeth)” and “showered”, where the most reported words include “tv” (*N* = 201), “ate” (*N* = 173) and “watched” (*N* = 152). The words significantly related to activities having the greatest effect on the participants’ SWB in the past four weeks (the red words to the left) include many words related to social activities (e.g., “family”, “friends” and “socializing”) and physical activities (e.g., “walking”, “football” and “exercise”), where the most reported words include “walking” (*N* = 60), “family” (*N* = 59) and “shopping” (*N* = 59). The grey words significantly belong to one activity category but nevertheless occur more frequently in the other category; thus, the word embedding of “tv”, “shopping” and “work” significantly relate to activities with the greatest effect on SWB over the past four weeks, but the words occur more frequently in Yesterday’s Activities. The most frequent words not significantly belonging to any of the activity categories (in black) include many food-related words, e.g., “lunch”, “dinner” and “breakfast”. The words in the plot show the difference between activities reported for Yesterday’s overall Activities and for the activities with the greatest effect on their well-being the past four weeks.

## Discussion

Hypotheses 1a and 1b were that individuals’ self-reported activities for yesterday and their predicted valence would be significantly associated with SWB, but these hypotheses were not corroborated. Previous studies finding significant associations between activities and SWB have explored pre-defined activities and their relationship with SWB [11, 15] or used an experience sampling method assessing overall everyday activities and focusing on momentary SWB [8, 29]. We asked about the overall activities of one day (yesterday) in an open-ended manner rather than pre-defining the activities, and asked the questions retrospectively instead of at random moments over a course of several days. We then correlated these answers with overall SWB. It is notable that participants’ responses were, for the most part, ordinary activities that most people do, including “work”, food-related activities such as “ate” and “drank’’, and home-related activities such as “brushed (teeth)” and “cooked” (treatment and meaning of verb tense is discussed below). These ordinary activities probably have nothing or little to do with SWB in a normal population, especially since it seems like these activities do not capture any activity features, like dosage or variation.

H2a hypothesized that the self-reported activities having the most impact on SWB the past four weeks would predict SWB, and this hypothesis was also not generally corroborated. The training of these activities to SWB was only significant for PA, whereas the predicted valence of these activities (H2b) significantly correlated with all of the SWB measures. Training word embeddings to predict a numerical scale is more dependent on sample size than correlating with predicted valence, since the valence model is created by another dataset. The correlations of the training in this study thus might have been higher if we had used a bigger sample. The word plot suggests that words related to social and physically active activities such as “family”, “walking”, “friends” and “football” relate to activities that individuals report to affect SWB, but do not in this analysis indicate whether the impact was positive or negative.

Fig 1 reveals that participants mainly used the past tense to describe Yesterday’s Activities and present participles to describe activities performed in the past four weeks. The difference in verb tense makes it possible for the same word stem to appear on both sides of the plot and thus “cook(ing)ed”, “dr(iving)ove”, “walk(ing)ed” and “stud(ying)ied” can be interpreted as common activities yesterday that participants perceive have affected their well-being during the past four weeks. However, the direction of the impact of these activities remains unclear.

The question regarding activities that most affected SWB was inspired by questions that generated strong correlations to SWB when participants were asked to report words reflecting their Harmony in Life or Satisfaction with Life [31]. However, the participants in Study 1 were asked to report activities with the most *impact* on their SWB, rather than activities *reflecting* their SWB. In addition, Study 1 did not explicitly examine whether different individuals understand how their activities relate to their SWB in the same way.

## Study 2

To further examine how or whether individuals understand the link between their everyday activities and SWB, Study 2 examines individuals’ views of 1) activities they regularly do that decrease or increase their SWB, and 2) activities they regularly do that *reflect* their overall SWB. In other words, we examine whether individuals have a shared understanding about which activities decrease and increase SWB, and provide descriptions of these activities. If individuals tend to have considerably different understandings of what activities decrease versus increase SWB, this result could explain the rather low correlations between activities and SWB measures in Study 1. That is, if individuals show low agreement, it means, for instance, that one individual thinks that studying for an exam decreases SWB, while another individual thinks that studying for an exam increases SWB. Predicting SWB scores from these activities is difficult without knowing more about the individuals. We will test this level of agreement in Study 2.

If individuals share an understanding about activities that decrease versus increase SWB, this common ground would enable us to make more accurate SWB predictions from the answers to the second question, where individuals are asked to write activities reflecting their SWB. However, being able to predict accurate SWB scores from the question not only requires individuals to share an understanding of which activities decrease and increase SWB, but it also requires individuals to understand the link introspectively. That is, individuals could understand that “playing football” tends to be an activity that increases one’s SWB, but not understand that the activity *reflects* one’s overall SWB.

Furthermore, in Study 2, individuals are able to report their activities using multiple words for the activities increasing/decreasing SWB rather than being encouraged to use one or two words, as they were in Study 1. Allowing more words has the potential to capture activity features; for instance, individuals have the option to add dosage to an activity, such as reporting “working *too much*”.

The assessment of individuals’ understanding of how their everyday activities link to their SWB is slightly different from Study 1 in two ways. First, we ask for *regular* activities instead of activities during *the past four weeks* in order to capture everyday activities accurately. Second, we ask about activities *reflecting* SWB instead of *impacting* SWB. The word “impact” implies a change in SWB, while “reflecting” implies an assessment of the current level. In addition to allowing open-ended answers for the activity questions, we further extend the method of measuring SWB in order to also enable individuals to describe their SWB with words using open-ended answers [32].

The hypotheses of Study 2 include:

### Individuals share an understanding of what activities decrease or increase SWB

#### Hypothesis 3

Individuals agree about activities that decrease SWB versus increase SWB. This hypothesis is tested quantitatively by training word embedding as a decreasing or increasing response and qualitatively by examining the word plots differentiating the two. Activities perceived as increasing SWB are hypothesized to relate to social and physically and cognitively active activities, reflected in words like “family”, “friends”, “walking” and “gym”, whereas activities perceived as decreasing SWB are expected to relate to passive activities, such as “TV” and ordinary everyday activities in the form of duties, such as “cleaning” and “work”.

### Activities reflecting SWB relate to SWB (three tests)

Whether activities reflecting SWB relate to SWB is tested using three different methods including i) directly training word embeddings to the SWB scores, ii) predicting valence scores from the word responses and correlating them with SWB, and iii) applying SSS to the activities reflecting SWB word responses to both the activities increasing and decreasing SWB responses, and correlating them with SWB.

#### Hypothesis 4a

Activities that reflect SWB significantly predict SWB measures.

#### Hypothesis 4b

The predicted valence of the activities that reflect SWB correlate positively and significantly with the SWB measures. There will be a negative correlation with NA.

#### Hypothesis 4c

The Bipolar SSS between activities reflecting SWB and the respective word norms for activities increasing minus decreasing SWB will positively and significantly correlate with the SWB measures. There will be a negative correlation with NA.

### SWB word descriptions predict SWB scores

As a replication of previous studies [31, 32], we hypothesize that the SWB word responses will predict numerical SWB.

#### Hypothesis 5a

Harmony in Life word responses will significantly predict HILS-3 scores.

#### Hypothesis 5b

The predicted valence of Harmony in Life word responses will significantly correlate with HILS-3 scores.

#### Hypothesis 5c

Satisfaction-with-Life word responses will significantly predict SWLS-3 scores.

#### Hypothesis 5d

The predicted valence of Satisfaction with Life word responses will significantly correlate with SWLS-3 scores.

## Method

### Participants

Three hundred and one adults in the UK were recruited through Prolific. Five failed to give their Prolific ID and three failed to answer a control question, and thus a total of eight were excluded from further analysis, leaving 293 participants. Participants were compensated £0.8 to participate in the study, which took on average 9.88 minutes (*SD* = 5.46) to complete. Of the 293 participants, 225 were female and 68 males with a mean age of 35 (*SD* = 12, range 18–75) years. Regarding employment status, 125 worked full-time, 79 worked part-time, 18 were unemployed and 71 had some other employment status, such as non-paid work. Seventy participants were students. The average subjective socioeconomic status was 5.25 (*SD* = 1.44).

### Instruments

#### Activities increasing and decreasing SWB

Participants were asked to write which activities they regularly do that increase or decrease their overall SWB in two separate questions. The assessment allowed open-ended answers to the instruction “Write 5 activities that you regularly do that increase (*or* *decrease*) your overall well-being.” Participants were required to report five activities on each of the two questions.

#### Activities reflecting SWB

Participants were asked to write the five activities they regularly do that best reflect their overall SWB, assessed by allowing open-ended answers to the instruction “Write the 5 activities that you regularly do that best reflect your overall well-being. The activities can have a positive or negative impact on your own overall well-being” along with the following instruction: “Please answer the question by writing the 5 activities you regularly do that best reflect your overall well-being. Try to weigh the amount of activities having positive or negative impact on well-being so that they reflect your overall well-being. For example, if you experience high well-being, write more activities you regularly do that best reflect this, and if you experience low well-being, write more activities you regularly do that best reflect that. Write descriptive words relating to those activities that are most important and meaningful to your overall well-being. Write only one descriptive word in each box.”

#### Bipolar SSS for activities

We created norms for activities that increase/decrease SWB by the participants’ responses. We subtracted 1) the SSS between the activities reflecting SWB and the norm for activities decreasing SWB, from 2) the SSS between the activities reflecting SWB and the norm for activities increasing SWB. The difference scores create the variable bipolar SSS for SWB-related activities (henceforth called *bipolar SSS for activities*).

#### Subjective Well-Being assessments

The numerical SWB scales were the same as used in Study 1, namely the HILS-3, the SWLS-3, the PA, and the NA scales from the PANAS and the SWB composite. All scales yielded a Cronbach’s alpha and McDonald’s omega between .91–.95. The control items used in Study 1 were also used in Study 2.

Study 2 included computational language assessments of Harmony in Life and Satisfaction with Life (as developed by [32]). Participants were asked to answer the following question: “Overall in your life, are you in harmony or not?” using five words, and adapted to reflect the amount of satisfaction. Participants were instructed to report five descriptive words depicting their harmony and satisfaction. These answers and their predicted valence were used as four of nine SWB measures. The valence was computed with the same valence model as in Study 1.

Bipolar SSS for Harmony in Life (HIL) and Satisfaction with Life (SWL) were used in Study 2. The SSS between the HIL/SWL word responses and the disharmony/dissatisfaction norms [31] were subtracted from the SSS between the HIL/SWL word responses and the harmony/satisfaction norms [31], computing the *bipolar SSS for HIL* and the *bipolar SSS for SWL*, respectively. These two variables constitute two of the nine SWB measures.

### Procedure

The recruitment process for Study 2 participants was identical to Study 1, and those who participated in Study 1 could not participate in Study 2. Participants began the survey by giving open-ended answers about activities they regularly do that increase/decrease their overall SWB, and next provided open-ended answers about the activities they regularly do that best reflect their overall SWB, and finally were presented with the open-ended questions regarding HIL and SWL in randomized order. Then, the participants answered the numerical SWB scales, randomized as in Study 1. All data was collected between 5 pm and 7 pm on March 13th, 2020.

### Ethical statement

The ethical procedure reported for Study 1 also applies to Study 2.

## Results

All numerical scales show normality with skew scores ranging from -0.61 to 0.57 and kurtosis scores ranging from −0.8 to 0.19 and were analyzed with Pearson correlation. The SWB rating scales correlate strongly to each other (significant correlations ranging from -.53 to .83, all *p*-values < .001, except for a moderate correlation between PA and NA (*r* = -.44, *p* < .001)). The correlations are corrected for multiple comparisons using Holm correction [46]. All numerical SWB correlations and descriptive statistics are found in Tables S2.1 and S2.2 in S1 File.

### Activities increasing and decreasing SWB plotted

**Fig 2:**
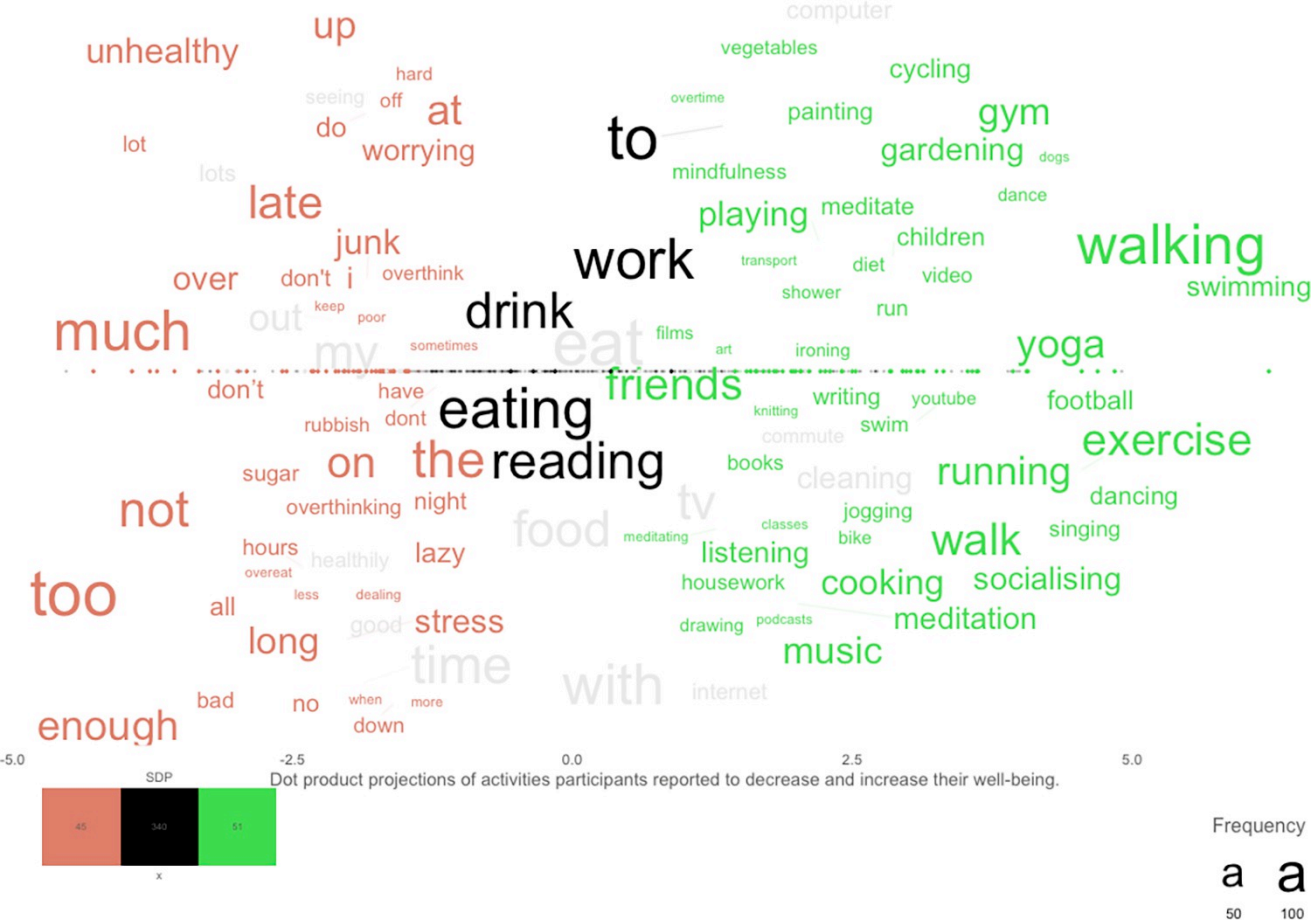
Words for describing activities that decrease and/or increase Subjective Well-Being. *Note*: Supervised dimension projection plot for activities that increase and/or decrease SWB. Words appearing a minimum of 5 times in the questions combined have been significance tested against a permuted null distribution, *N*_*permutations*_ = 200,000. Words significantly belonging to activities increasing SWB are plotted on the right side in green. Words significantly related to activities decreasing SWB are plotted on the left side in red. Black words in the middle are frequent words that do not significantly belong to any group. Grey words significantly belong to the activity category of its position, but occur more frequently in the other category. Word size represents frequency. The position on the dimension projection and the position on the x-axis represent the dot product score. For better visualization, the words are separated on the y-axis, but the y-axis does not represent any information.

To examine which everyday activities individuals relate to well-being, activities that increase and/or decrease SWB are plotted in Fig 2 in a supervised dimension projection plot. The words significantly related to activities that decrease SWB (the red words to the left) include many function words and adverbs, such as “enough”, “late”, “too”, “much” and “unhealthy”. The words significantly related to activities that increase SWB (the green words to the right), include many words relating to active physical activity (e.g. “football”, “swimming” and “dancing”), but also words relating to cognitively active activities (e.g. “meditation”, “mindfulness”, “listening”) and social aspects of activities (e.g. “socialising”, “friends”, “children”). Words significantly belonging to one or the other of the SWB categories (i.e., increase or decrease), but that nevertheless occur more frequently in the opposite category, include “tv”, “computer” and “good” (the words in grey). Common words that do not significantly belong to any of the groups (black words) include descriptions of ordinary everyday activities, such as “eating”, “work” and “drink”. A list of the words with highest and lowest dot product projections is found in Table S2.3 in S1 File.

### Individuals share understandings about activities that decrease and/or increase SWB

The word embeddings of the activities that decrease and/or increase SWB were trained using logistic regression to classify them as either an SWB-decreasing or SWB-increasing activity. The model yields an AUC of .995 with a balanced accuracy of .968. The same analysis was done with every word embedding of all single activity responses. The logistic regression model yields an AUC of .964 with a balanced accuracy of .902. The results indicate that participants strongly agree about how activities relate to SWB and confirm H3. A strong agreement is necessary if individuals’ descriptions of their SWB, through activities, are to predict their SWB at the group level.

### Activities predict SWB

The next analysis examines whether individuals can describe how their everyday activities relate to their SWB. This test is made by predicting rated SWB from the everyday activities individuals perceive to reflect their SWB. The activities reported to reflect SWB, their predicted valence, and the bipolar SSS for activities generally yield significant correlations with the SWB measures (Table 3). The predicted SWB scores from the word embeddings of the activities reflecting SWB significantly but weakly correlate to all SWB measures (significant correlations ranging from .15 to .26, all *p*-values < .05), except for the BSSS for SWL.

**Table 3:** Relationship between activities reflecting SWB and various SWB measures.

	Activities reflecting SWB		
Variables	Training^1^	Valence^2^	Bipolar SSS
**HILS-3**	.22**	.28**	.17**
**Bipolar SSS for HIL**	.19**	.27**	.19*
**HIL Valence**	.15*	.26**	.10
**SWLS-3**	.19**	.20**	.14
**Bipolar SSS for SWL**	.10	.21**	.17*
**SWL Valence**	.18**	.27**	.13
**PA**	.17**	.30**	.14
**NA**	.26**	-.29**	-.19**
**SWB**	.25**	.33**	.19**

*Note*. N = 293

* *p <* .*05*

** *p* < .01 (2-tailed), Holm corrected for multiple comparisons. Valence = predicted valence of text responses, HILS-3 = Harmony in Life Scale three item version, SWLS-3 = Satisfaction with Life Scale three item version, PA = Positive Affect, NA = Negative Affect, SWB = Subjective Well-Being composite score.

^1^ = Activities reflecting SWB were trained to predict the scales.

^2^ = Predicted valence of the activities reflecting SWB.

A similar pattern is found regarding the predicted valence of the activities reflecting SWB: these values correlate with the SWB measures in the expected direction. The range of the correlations is slightly higher for valence than training the activities reflecting SWB to the SWB measures; valence correlations range from .20 to .33 (negatively at *r* = -.29 for NA, all *p*-values < .01). The correlations are weak, except for PA and SWB, which correlate moderately.

Bipolar SSS for activities correlates significantly with five of the nine SWB measures. The HILS-3, the bipolar SSS for HIL, the bipolar SSS for SWL, the NA, and the SWB composite yield small significant correlations in the expected direction to the bipolar SSS for activities, ranging from *r* = .17 to .19 (negatively at r = –.19 for NA). This result means that participants with a high SWB describe their activities that reflect their SWB differently from those with a low SWB. Generally, these results support hypotheses 4a, 4b, and 4c and provide an (affirmative) answer to the question of whether individuals can describe how their everyday activities relate to their SWB. Plots depicting the most central words for activities reflecting SWB and plots of these words to reported SWB are found in Figs S2.1–2 in S1 File.

### Participants can describe their SWB with words that predict their SWB rating scales score

In accordance with previous studies and H5a–d, the HIL words, and the SWL words (including their predicted valence) generally correlate strongly to all numerical SWB scales (Table 4) with *r* ranging from .39 to .61 (all *p* < .01). This result means that the participants’ descriptions of their SWB strongly relate to their SWB rating score.

**Table 4:** Relationship between HIL and SWL words and numerical SWB scales.

Word responses	Method	SWB	HILS-3	SWLS-3	PA	NA
**Harmony**	Training	.55	.58	.49	.39	-.43
**words**	Valence	.56	.59	.50	.42	-.40
**Satisfaction**	Training	.63	.58	.60	.51	.49
**words**	Valence	.63	.61	.60	.45	-.51

*Note*. N = 293. All *p* < .001 (2-tailed), Holm corrected for multiple comparisons. Training = predicted rating scale scores from text responses using Ridge Regression, Valence = predicted valence of text responses, HILS-3 = Harmony in Life Scale three item version, SWLS-3 = Satisfaction with Life Scale three item version, PA = Positive Affect, NA = Negative Affect, SWB = Subjective Well-Being composite score.

## General discussion

### Activities that decrease versus increase SWB

The results of Study 2 demonstrate that individuals show strong agreement over which activities they relate to decreasing versus increasing SWB, in line with H3 of Study 2. The logistical regression model categorizes the participants’ full answers with exceptionally high accuracy and the model for the individual activities with very high accuracy as either increasing or decreasing SWB activities. The results demonstrate that, overall, individuals share an understanding about which activities decrease or increase their SWB.

The word plot depicting these activities shows clearly that the activities that increase SWB yield many activities that has be demonstrated in previous research, including many physically active activities (e.g. “running”), cognitively active activities (e.g. “mindfulness”), and social (e.g. “socialising”) activities. Some words are not activities themselves but rather related to activities, such as “friends” and “books”. Words related to decreasing SWB include very few activities per se, but instead predominantly include descriptions of imbalance through the activity features dosage (e.g., “late”, “not” and “enough’’) and (low) variation (e.g., “too”, “much” and “long”). These results also align with previous research, especially the Positive-Activity model [11], where activity features, including dosage and variation, play a fundamental role in the relationship between activities that increase SWB and SWB. Interestingly, activity features mostly relate to decreasing SWB activities in our sample, which might speak to a generally positive attitude toward activities as long as they are carried out in moderation. Notably, the words describing activities with the most impact on SWB in the past four weeks (on the left side of Fig 1) as well as the centrality plot for activities reflecting SWB (Fig S2.1 in S1 File) align almost exclusively with activities that increase SWB (mainly social and physically active activities). It is possible that, when people are asked to report activities that explain SWB, activities that increase SWB are more easily described and/or more easily come to mind than activities that decrease SWB. Furthermore, it is possible that activities that increase well-being are less dependent on activity features (such as a dosage specification; see Fig 2)

### Describing SWB through activities and words

Predicting SWB from respondents’ descriptions of everyday activities reflecting their SWB (H4) resulted in both non-significant and significant correlations, with small to medium effect sizes. The predicted valence of the activities reflecting SWB shows stronger correlations than training the activity responses to SWB measures, as in Study 1 (in relation to activities with the most impact on SWB in the past four weeks correlated to SWB). The bipolar SSS for activities demonstrated the lowest correlations. Predicting SWB from word descriptions of SWB, on the other hand, including Harmony in Life and Satisfaction with Life, resulted in significant correlations to SWB scores with strong effect sizes, which agrees with H5a–d as well as previous research (e.g., [32]).

The overall results of the tests of H4 indicate that individuals understand the relationship between their activities and their SWB to some extent. The results further suggest that asking individuals about activities reflecting their SWB predicts SWB better than asking about which activities had the most impact on their SWB in the past four weeks, as was the case in Study 1. The correlations are, however, relatively small when considering how well individuals generally agree on what constitutes an activity that decreases or increases SWB, as well as predictions from individuals’ explicit descriptions of their SWB.

### The well-being/activity description gap

Notably, individuals are less accurate when describing their SWB with their activities (H4) than they are when freely describing their SWB with words (H5). SWB words predict SWB scores with correlations ranging from .39–.63, whereas activity words only predict SWB scores with correlations in the range of .10–33. We refer to this correlation gap as a *well-being/activity description gap*.

The gap’s relatively large size might be somewhat surprising and might suggest that individuals have a poor understanding of the link between their own activities and their overall SWB. To some extent, this gap is expected considering that describing SWB through words allows the answers to explicitly describe what SWB is, whereas describing SWB through activities constrains the responses to describe particular activities. At the same time, one could argue that the gap ought to be small if individuals had a good understanding of the link between their activities and SWB considering 1) the individuals’ strong agreement regarding what constitutes SWB-increasing versus SWB-decreasing activities (H3) and that 2) their understandings tend to align with research on the topic (e.g., physically active activities have been associated with high SWB [6, 8, 28, 29]). However, even if individuals agree about which activities increase versus decrease their SWB separately, they seem to possess a poor understanding of how their activities link to their overall SWB. Understanding all SWB nuances through activities (describing overall SWB through activities) appears more difficult than simply understanding one pole of SWB (describing activities that increase or decrease SWB).

### Combining activity features with ordinary everyday activities

Ordinary everyday activities together with activity features seem to play a substantial role among the activities that decrease SWB. The significant words describing activities that decrease SWB almost exclusively included activity features (e.g., “late”, “rubbish”, “too”, “much” and “not”; the left side of Fig 2) and the activities mentioned in combination with these activities often include ordinary everyday activities relating to food, sleeping, and work. This trend is illustrated by the words most frequently mentioned among the activities that increase versus decrease SWB that do not significantly belong to either increasing or decreasing SWB (the words in black in Fig 2; e.g., “eating”, “drink”, “work”) and among Yesterday’s Activities (the right side of Fig 1; e.g., “ate”, “slept”), which did not significantly relate to SWB, possibly because features were not assessed there. Combining these ordinary everyday activities with the activity features depicted among the activities that decrease SWB in Fig 2 (e.g., “work late” and “eating too much rubbish”) illustrates that balance among ordinary everyday activities is important for understanding activities that decrease SWB.

### Limitations and future studies

These two studies highlight the importance of considering response format when asking open-ended questions. One limitation of our interpretation of the results is that the response format of the question regarding activities that affected SWB the most in the past four weeks encouraged one word per response-box, and the question regarding activities reflecting one’s SWB required one word per response-box. The questions regarding activities that increase or decrease SWB allowed several words in each response-box and the results (Fig 2) suggest that describing SWB-related activities requires several words, especially concerning activities that decrease SWB. The format of “one word per response-box” achieved strong predictive results in previous studies when describing SWB with words [31], which is why we selected the limit. However, future studies should enable individuals to use several words when describing activities reflecting their SWB, which could potentially decrease the well-being/activity description gap.

The research in these studies asked for self-reported activities, which of course might not accurately reflect the activities that the participants truly did. Applying more elaborate research designs, such as the experience sampling method to more reliably measure activities, can potentially yield more insights into the relationship between actual activities and SWB. Assessing activities objectively could explain how large the gap is between the strength of the relationships between, on the one hand, objective activities and SWB, and on the other hand, individuals’ subjective experiences of the relationship.

Considering various SWB aspects, future research may use this computational language assessment method to examine different dimensions of well-being (such as happiness) by asking individuals to describe their happiness in activities and words, and explore how describing happiness affects the well-being/activity description gap. Furthermore, it is also possible to ask individuals to report specific categories of activities (such as focusing on work and duties, or focusing on reporting activities carried out in their free time).

Individual understandings of the link between SWB and activities could be further explored by accounting for personal differences. For example, individuals with different levels of personality traits likely prefer different types of activities as well as different dosages of the same activity, even if they agree on which activities increase versus decrease SWB. Extraverts and introverts might agree that different social activities, such as “partying”, increase SWB, but extraverts may need a greater dosage of “partying” to reflect high personal SWB. Here, individuals would need to indicate activity features if the activities are to reflect SWB. Taking individual differences such as personality into account could disentangle whether the ability to understand the link between one’s activities and SWB is poor or if such other factors explain the low correlation.

Future studies could also test whether interventions targeted at making individuals think more about how activities reflect the SWB of individuals, decrease the well-being/activity description gap and subsequently increase individuals’ SWB. The description gap suggests that individuals might not fully understand how the activities they do reflect their SWB. However, the gap could have been larger, considering the significant (but quite small) correlations between activities reflecting SWB and the SWB measures, and the fact that thinking about activities that reflect one’s SWB is probably not a standard practice of most people. Agreement among individuals about activities that increase and decrease SWB and corroboration by previous research suggests that it might be possible to make individuals better understand what makes them happy and subsequently increase their SWB.

Future studies could also investigate how everyday activities relate differently to momentary versus overall SWB using the proposed NLP approach. This could be done by combining the experience sampling method and the retrospective method used in this study. Some previously reported activities with a strong relation to momentary SWB (using experience sampling methods), such as “Sex” and “Partying” (both *N* = 1 on activities reflecting SWB [8]), were mentioned a few times in our studies in the context of overall SWB. These examples of activities relating differently to momentary and overall SWB suggest a potential difference in how activities relate to momentary versus overall SWB, which should be further explored.

## Conclusion

We investigated the relationship between self-reported everyday activities and SWB, and especially individuals’ own understandings of the relationship between them. To assess activities, we allowed open-ended answers that let individuals describe their everyday activities freely, which were analyzed quantitatively with state-of-the-art NLP techniques. The results demonstrate that individuals show a strong agreement regarding which activities they consider to increase versus decrease their SWB. SWB increasing activities (as reported by the participants) included social activities and physically and cognitively active activities, whereas activities that decrease SWB were described (by the participants) predominantly with words about ordinary everyday activities but performed in an imbalanced way; these words related to activity features, such as dosage and variety.

Despite the strong agreement about what activities individuals consider to increase versus decrease SWB, there were only small to medium relationships between reported activities reflecting a participant’s personal SWB level and their SWB scores, whereas their SWB descriptions with words showed a strong relationship to their SWB scores. This well-being/activity description gap may be interesting to further examine in future research. The method provides new perspectives concerning the relationship between SWB and activities. Specifically, NLP can be used to analyze everyday activities in relation to SWB, which might be interesting because individuals show a relatively poor understanding of how their everyday activities relate to their SWB, and of how activity features play a role (especially in relation to decreasing SWB). Considering the great value most people put on SWB and that we fill our lives with activities, the NLP method and these findings are important for future research as well as from the perspective of individual humans.

### Data availability statement

Study 1 and Study 2 were both pre-registered at the Open Science Framework (osf.io). The data and code are available here.

## Supporting information

S1 File (DOCX)
