# Supplementary material for: Doing well-being: Self-reported activities are related to subjective well-being
Source: PLoS One. 2022 Jun 24;17(6):e0270503. doi: 10.1371/journal.pone.0270503 (PMC9232132; doi:10.1371/journal.pone.0270503)
Supplement: S1 File — (DOCX) [file pone.0270503.s001.docx]

**Doing Well-Being: The Role of Self-Reported Activities in Relation to Subjective Well-Being**

**Supporting Information**

The supplemental material begins with a cohesive examination of the pre-registered hypothesis in Study 1 regarding variation in activities and SWB. After this examination are the remaining supplemental material of Study 1, including pre-registered analyses in Semantic Excel, followed by the supplemental material for Study 2

**Contents**

1. **Variation in activities, Study 1 2**

Method 2

Results 3

Discussion 3

1. **Remaining Material Study 1 4**

Results 4

The relationship between self-reported activities and SWB scales 5

Sections of the day in relation to SWB 5

Semantic coherence of the different sections of the day 6

Word plots Semantic Excel 6

Discussion 12

1. **Study 2 13**

Descriptives of SWB scales 13

Intercorrelations among SWB scales 14

Unipolar SSS and activities reflecting SWB 15

Word plots 16

**Variation in Activities, Study 1**

Besides the hypotheses in the main article, Study 1 also explored if variation in activities is related to SWB through the following hypotheses:

**Hypothesis 3a.** The number of activities participants report they did yesterday correlates with their SWB (positively with HIL, SWL and PA, and negatively with NA).

**Hypothesis 3b.** The variation in the activities reported yesterday as measured by 1) the coherence of the words describing the activities and 2) participants’ subjective perception of variation, correlates with SWB (for coherence, negatively with HIL, SWL and PA, and positively with NA, and the inverse for participants’ subjective perception of variation).

**Method**

***Measure of variation in activities.*** A three-item measure of perceived variation in activities was developed in a recent study and used to measure subjective perception of variation in activities (Etkin & Mogilner, 2016). The items (e.g., “How much variety is there among the activities you did yesterday?”) were answered on Likert scales ranging from 1 (“Very little variety”) to 7 (“A lot of variety”). Cronbach’s alpha has previously been .90 (Etkin & Mogilner, 2016). In this study, Cronbach’s alpha was .73 and McDonalds omega total was .76.

***Semantic coherence.*** To capture the semantic coherence of word responses we measure the average SSS between consecutive words in a response. If a response contains three words, the semantic coherence is equal to the average of the SSS between the first and the second word, and the second and the third word. The consecutive method implies that “hot dog cat” will have a higher coherence than “hot cat dog”. Also, function words such as “the”, “in” and “at” are not excluded, thus “flying airplane” will have a higher coherence than “flying the airplane”. This is generally problematic; but might be seen as a good proxy in Study 1 considering that we are using descriptive words rather than sentences that include a lot of function words.

Similarly as predicting valence of words, one can predict arousal through the Affective Norms for English Words (ANEW; Bradley & Lang, 1999). The arousal model predicted valence of the ANEW words at *r* = .65 (*N*_words_ = 1,029, *p* < .001).

***Semantic Excel.*** The NLP analysis of Study 1 was pre-registered to be done in the online software Semantic Excel ([semanticexcel.com](http://www.semanticexcel.com); for an overview of the tool, see Sikström et al, 2018) using the semantic space referred to as “English 2” . This was due to the fact that the more advanced BERT model (being able to contextualise words and analyse sentences) was not easily accessible for social scientists. To make the analysis coherent, the NLP analysis of Study 1 was re-run with the BERT model in the R-package *Text*. The differences were small, but generally the results were more consistent using BERT, i.e., the correlations to the various SWB constructs were more coherent. The pre-registered analysis of Study 1 computed in Semantic Excel can be found here in the Supplemental material.

**Results**

**Variation in and quantity of activities in relation to the SWB scales**

Subjective variation in yesterdays’ activities had significant correlations (*Table S1.1*) to the HILS-3 (*r* = .15; *p* < .05), the SWLS-3 (*r* = .16; *p* < .05) and the PA scale (*r* = .14; *p* < .05); but no significant correlation to the NA scale (*p* > .05). The semantic coherence of the activities reported for yesterday as a whole yielded no significant correlation with any of the SWB scales (all *p* values > .05). Taken together, the results indicate partial support for hypothesis 3b. The number of activities reported for yesterday as a whole yielded no significant correlation with any of the SWB scales (*p* > .05), indicating no support for hypothesis 3a.

**Table S1.1. Pearson correlations between SWB scales and variation in and number of activities.**

**Variables HILS-3 SWLS-3 PA scale NA scale**

**Semantic Excel**

Number of activities .05 .02 -.01 .03

Subjective variation .15* .16** .14* -.01

Coherence yesterday .06 .01 -.04 .11

**BERT**

Number of activities .05 .02 -.01 .03

Subjective variation .15* .16* .14* -.01

Coherence yesterday .06 .01 -.04 .11

*Note*. N = 287 * *p* < .05; ** *p* < .01 (2-tailed). HILS-3 = Harmony in Life Scale three item version, SWLS-3 = Satisfaction with Life Scale three item version, PA = Positive Affect, NA = Negative Affect, Coherence = semantic coherence in the activities reported.

**Discussion**

Neither the number of activities nor semantic coherence of activities yielded any significant correlation with any of the SWB scales. However, subjective variation yielded weak correlations with three out of four SWB scales (the HILS-3, SWLS-3 and PA scale), which is in line with previous research (Etikin & Maligner, 2016; Sheldon et al., 2012). In the Positive Activity Model (Lyubomirsky et al., 2013), variation is seen as a feature of activities that is a positive factor for enhancing well-being, which our results indicate some support for.

The hypothesis regarding a correlation between number of activities and SWB, is based on the broaden-and-build theory (Fredrickson, 2001) and the subsequent finding that people induced into a state of positive emotions can think of more activities they would like to do than people in a neutral state (Fredrickson & Branigan, 2005), and that number of activities participated in have been correlated to SWB (Menec, 2003; Lawton et al., 1999). Also, a large number of activities indicates variation in activities, which relate to high SWB (Etkin & Maligner., 2016; Sheldon et al., 2012), further strengthening the hypothesis. However, there is no correlation between the number of reported activities and SWB. It is possible that the definition provided to the participants of what constitutes an activity was too broad. Since an activity was defined as virtually anything one can do, participants in this study could theoretically think of an infinite number of activities they had done, reducing activities down to even breathing and blinking. Indeed, typical answers for the yesterday question include “brushed”, “teeth” and “shower”.

Semantic coherence yielded no significant correlations with the SWB scales, weakening the association. However, this result is probably explained by semantic coherence being a poor measure of variation in activities, especially when participants can answer with two words (see method section here in the supplemental material on how coherence works).

**Remaining Supplementary Material Study 1**

***Wake up time and bedtime yesterday.*** Participants were asked to indicate at what time they woke up and went to bed yesterday by writing the time as four digits. Wake up time was used as a descriptive variable in the study in order to see if participants woke up at approximately the same time; since yesterday activities were divided into morning, day and evening activities, it was of interest to control that the participants have roughly the same circadian rhythm in order to make meaningful analyses. The average wake-up time was 07:29 (*SD* = 91.7 minutes) and average bedtime was 23.32 (*SD* = 94.7 minutes).

**Results**

**Table S1.2. Descriptive statistics for all the numerical variables.**

**Variables Mean *SD* Skew Kurtosis**

**HILS-3**  12.32 4.35 -.29 -.73

**SWLS-3** 12.16 4,48 -.36 -.78

**PA scale**  30.03 8.07 -.23 -.51

**NA scale**  20.64 8.01 .92 .31

**Subjective variation**  10.30 3.67 .15 -.46

**No. of activities yesterday** 14.22 6.20 .63 -.03

**Valence yesterday**  6.45 1.18 -.43 .44

**Valence past four weeks** 6.08 1.49 .06 -.05

**Coherence yesterday**  .07 .038 .93 1.82

*Note*. N = 295. HILS-3 = Harmony in Life Scale three item version, SWLS-3 = Satisfaction with Life scale three item version, PA = Positive Affect, NA = Negative Affect, Valence = predicted valence.

**The relationship between self-reported activities and SWB scales.** The results in *Table S1.3* are the same as the one in *Table 2* in the main article, but are here analysed with Semantic Excel.

**Table S1.3. Pearson correlations between semantic content prediction and observed SWB scale scores in Semantic Excel.**

**Variables HILS-3 SWLS-3 PA scale NA scale**

**Activities yesterday**  .00 -.02 .03 -.05

**Activities past four weeks**  -.01 .07 .04 .01

**Valence activities yesterday** .08 .07 -.03 -.13*

**Valence activities past four weeks** .11 .13* .10 -.11

*Note*. N = 295 * *p* < .05; ** *p* < .01 (2-tailed). HILS-3 = Harmony in Life Scale three item version, SWLS-3 = Satisfaction with Life scale three item version, PA = Positive Affect, NA = Negative Affect, Valence = predicted semantic valence.

**Sections of the day in relation to SWB.** Regarding the exploratory analyses of how well the different sections of yesterday (i.e., morning, day, evening) could predict SWB, morning had a small significant correlation to the *HILS-3* (*r* = .15, *p* < .01) whereas the rest of the SWB scales and parts of the day yielded no significant results, details in *Table S1.4*.

**Table S1.4. Trained predictions of how activities during different parts of yesterday predict SWB scales.**

**Variables Morning Day Evening**

**HILS-3**  .15** .01 .01

**SWLS-3**  .03 -.07 -.11

**PA** .03 -.10 .08

**NA** .01 .04 -.14

*Note*. N = 295. * = *p* < .05; ** = *p* < .01 (2-tailed). HILS-3 = Harmony in Life Scale three item version, SWLS-3 = Satisfaction with Life scale three item version, PA = Positive affect scale, NA = Negative affect, Morning = activities reported for yesterday morning, Day = activities reported for yesterday during the day, Evening = activities reported for yesterday evening. Computed in Semantic Excel.

**Semantic coherence of the different sections of the day.** Coherence morning and coherence day did not meet the pre-registered assumption of normal distribution (kurtosis > 2, see *table S1.5*) and were thus analysed with Spearman’s Rho. No correlations were found between semantic coherence of activities reported for the different sections of yesterday (morning, day and evening) and the SWB scales, as can be seen in *Table S1.6.*.

**Table S1.5. Descriptive statistics for coherence.**

**Variables Mean *SD*  Skew Kurtosis**

**Coherence Morning** .064 .054 1.619 5.075

**Coherence Day**  .07 .06 1.204 2.550

**Coherence Evening** .065 .038 .929 1.817

*Note*. N = 295. Computed in Semantic Excel.

**Table S1.6. Predicting SWB from semantic coherence for different sections of yesterday.**

**Variables HILS-3 SWLS-3 PA NA**

**Coherence Morning^** .05 .00 -.04 .00

**Coherence Day^**  .11 .05 .02 -.03

**Coherence Evening**  -.03 -.04 -.02 .06

*Note.* N = 295. * = *p* < .05; ** = *p* < .01 (2-tailed). ^ = Spearman’s rho, due to kurtosis > 2. Computed in Semantic Excel.

**Plots Semantic Excel.** *Figure S1.1* shows the most frequently answered words on the

activity question regarding yesterday. *Tv*, *watched*, *breakfast*, *lunch* and *dinner* were the most common activities for this question.

**Fig S1.1. Frequent words for Yesterday’s activities**


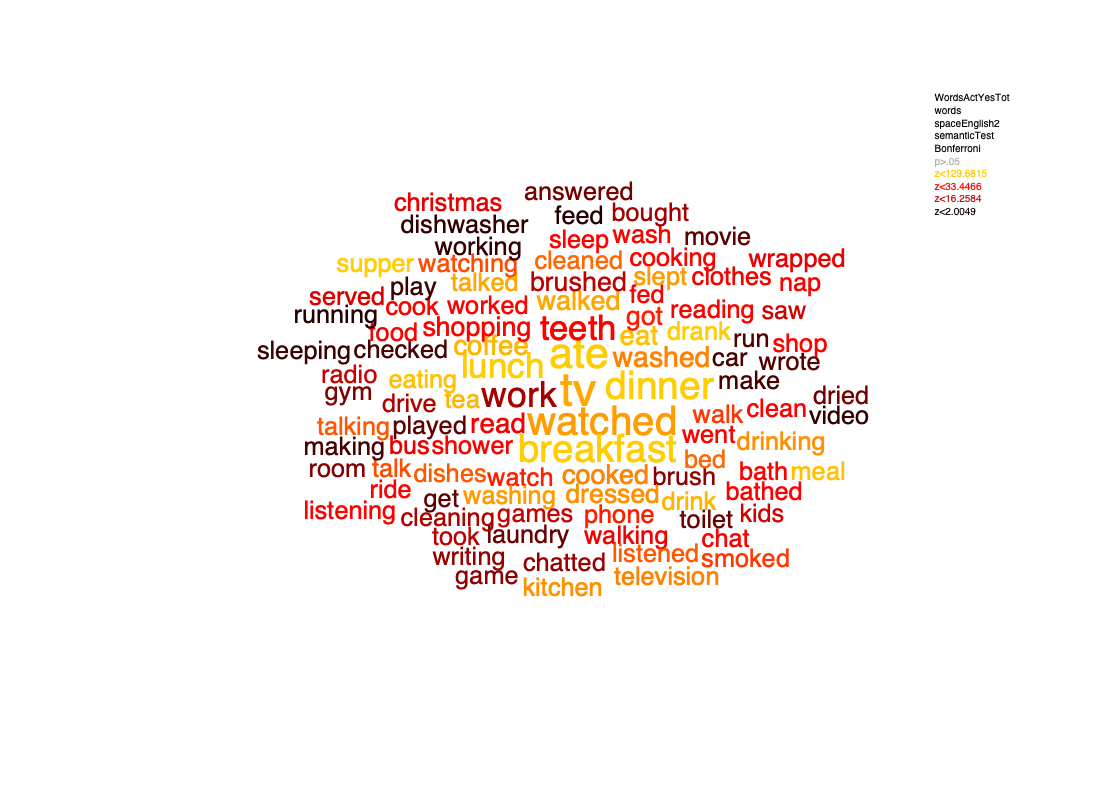


*Figure S1.1*. Yesterday’s activities. Semantic t-tests were computed where the words are compared to the frequency of how the words are generally used in the corpus *English 2*. Font size indicates frequency and colour indicates level of Z-transformed *t*-value. Z-values ranged from 129 to 2, darker colour indicates lower Z-value. All words were significant at *α* < .05. *r* = .69, *t* = 44.6, *p* < .01, Cohen’s *d* = 1.35.

*Figures S1.2a-d* show activities done yesterday plotted on the four SWB scales. *Breakfast*, *dinner*, *ate* and several words related to food were generally associated with high SWB in all scales. On the other hand, passive activities, and duties, such as *watched*, *washed* and *TV* were generally associated with low SWB.

**Fig S1.2. Yesterdays’ activities plotted along Subjective Well-Being dimensions**

*
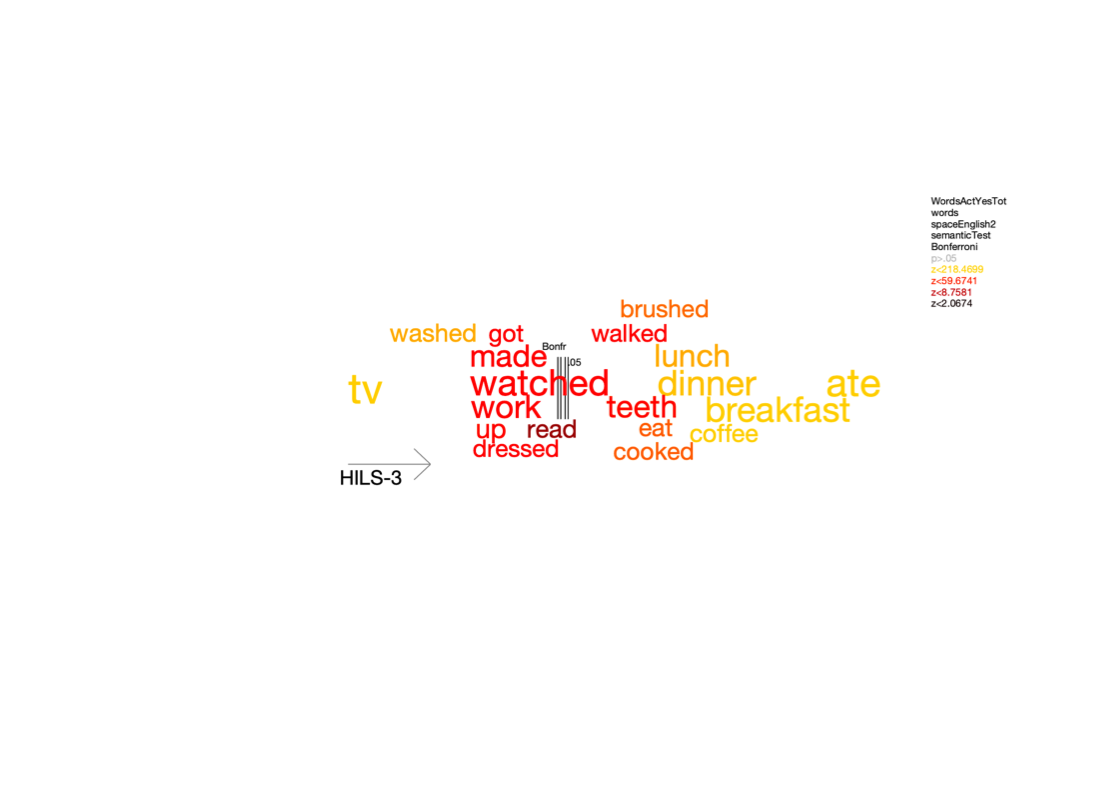

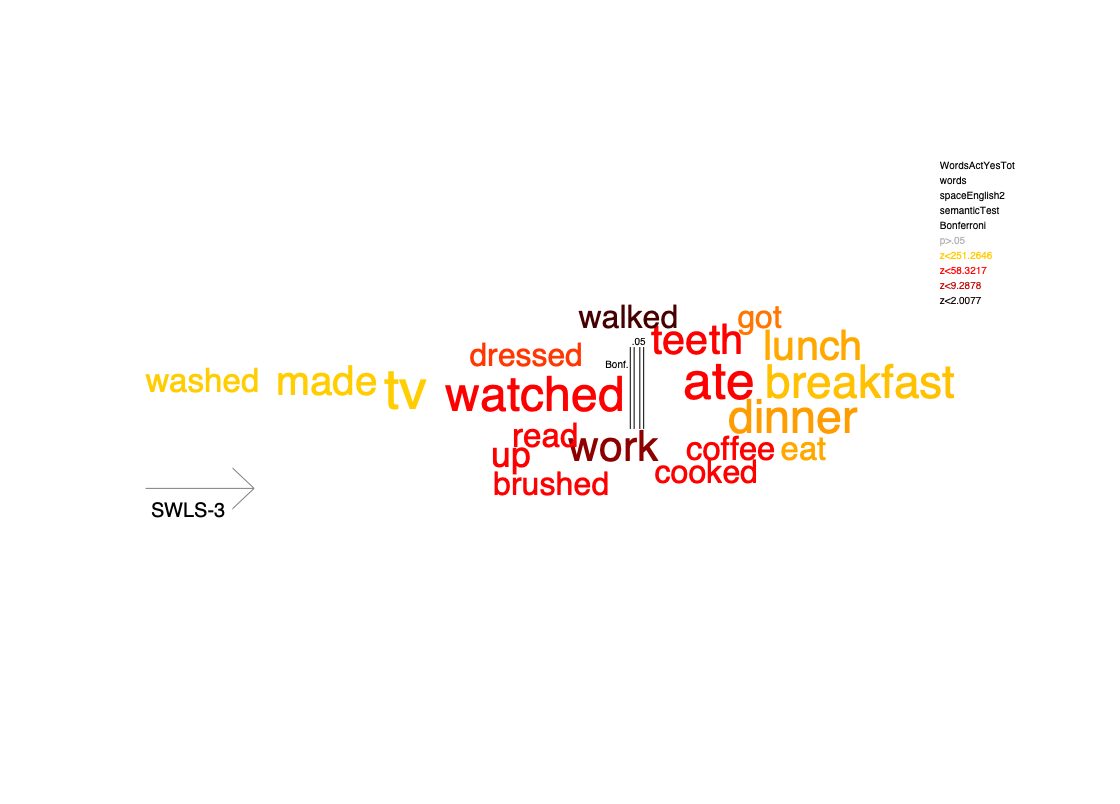
*

*S1.2a*. *r* = .11, *t* = 8.94, *p* < .01, *S1.2b*. *r* = .12, *t* = 9.75, *p* < .01 Cohen’s *d* =.16 Cohen’s *d* = .17

*
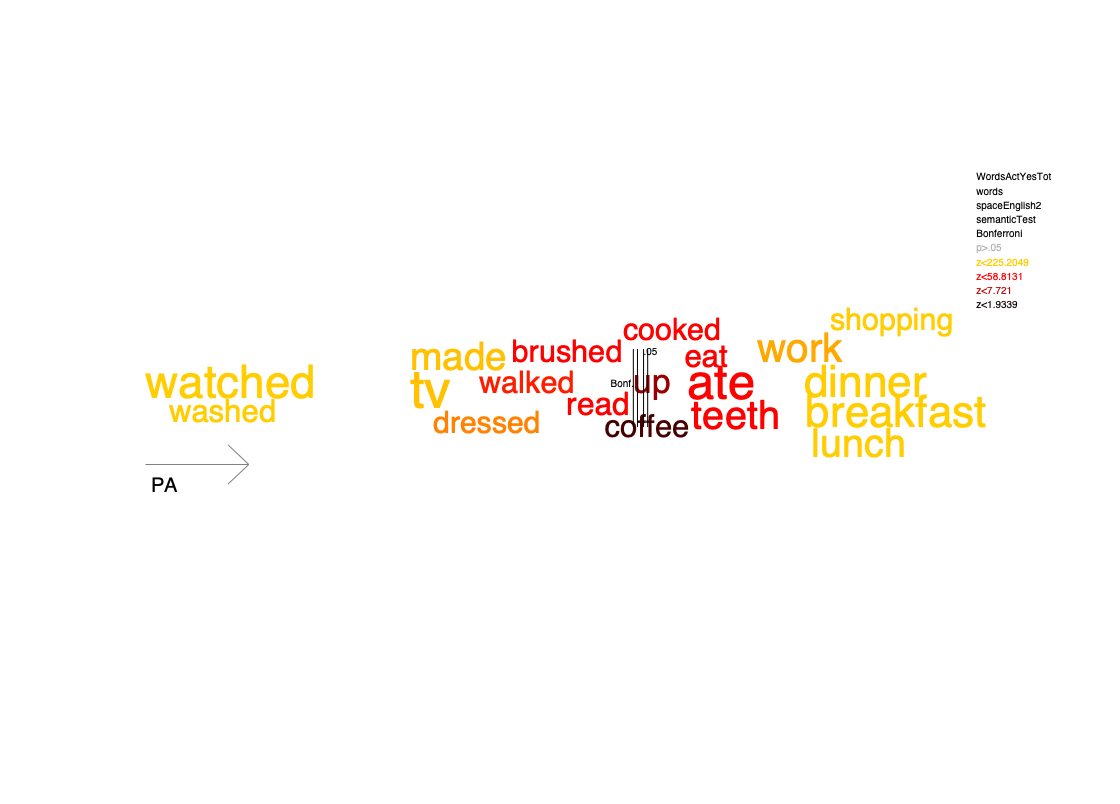

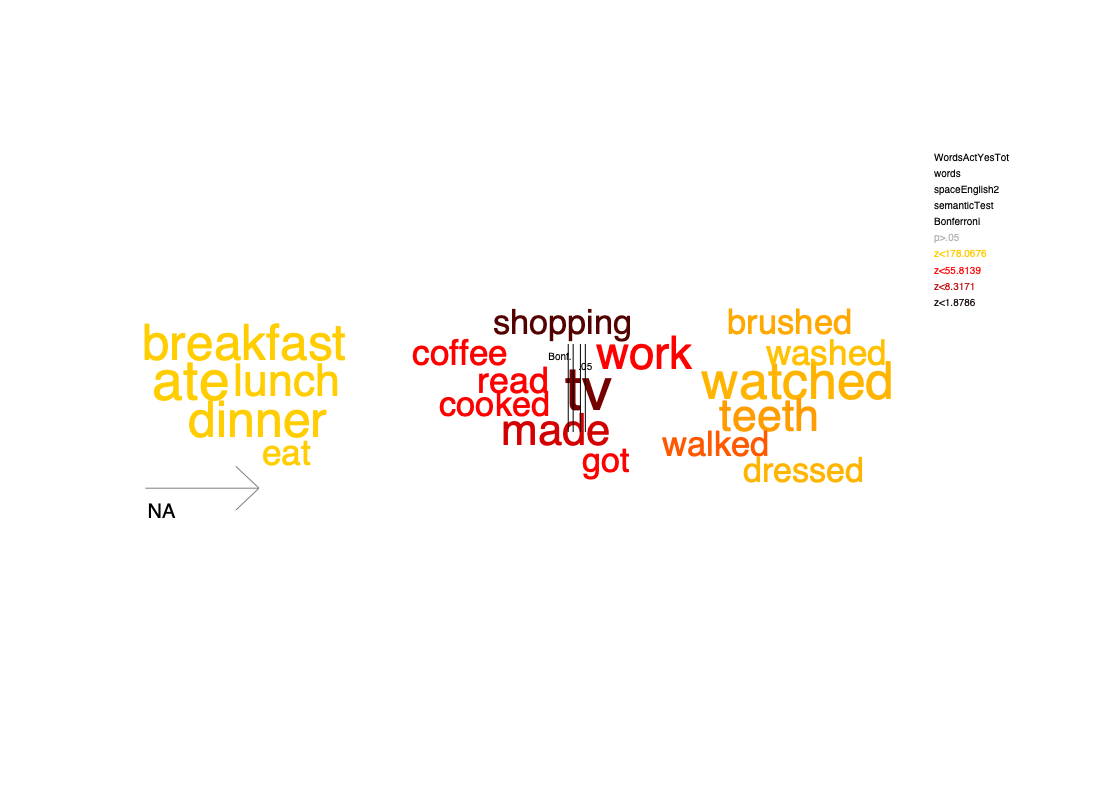
*

*S1.2c*. *r* = .14, *t* = 10.79, *p* < .01, *S1.2d*. *r* = .10, *t* = 8.18, *p* < .01, Cohen’s *d* = .19 Cohen’s *d* = .15

*Figures S1.2a-d*. Yesterday’s activities plotted on the four SWB scales. Significant keywords (*p* < .05) were plotted based on semantic *t*-tests for all individual words on the semantic content of yesterday's activities between high vs. low SWB scorers. The font size indicates frequency and colour the level of the z-transformed t-values. Z-values ranged from 178 to 1.9, darker colour indicates lower Z-value. Words farther away from the middle have higher Z-values. The plots show the 20 words with highest Z-scores. Plot *S1.2a*, HILS-3 = Harmony in Life Scale three item version, plot *S1.2b*, SWLS-3 = Satisfaction with Life scale three item version, plot *S1.2c*, PA = Positive Affect, plot *S1.2d*, NA = Negative Affect.

*Figure S1.3* shows the most frequently answered words on the activity question regarding the activities reported having the most impact on the participants’ SWB in the past four weeks. The most common activities were *family*, *walking*, *friends* and *reading*. *Figure 1* concerns word frequency only, and does not provide any information regarding if the impact on SWB is positive or negative.

**Fig S1.3. Activities having the most impact on well-being in the past four weeks**


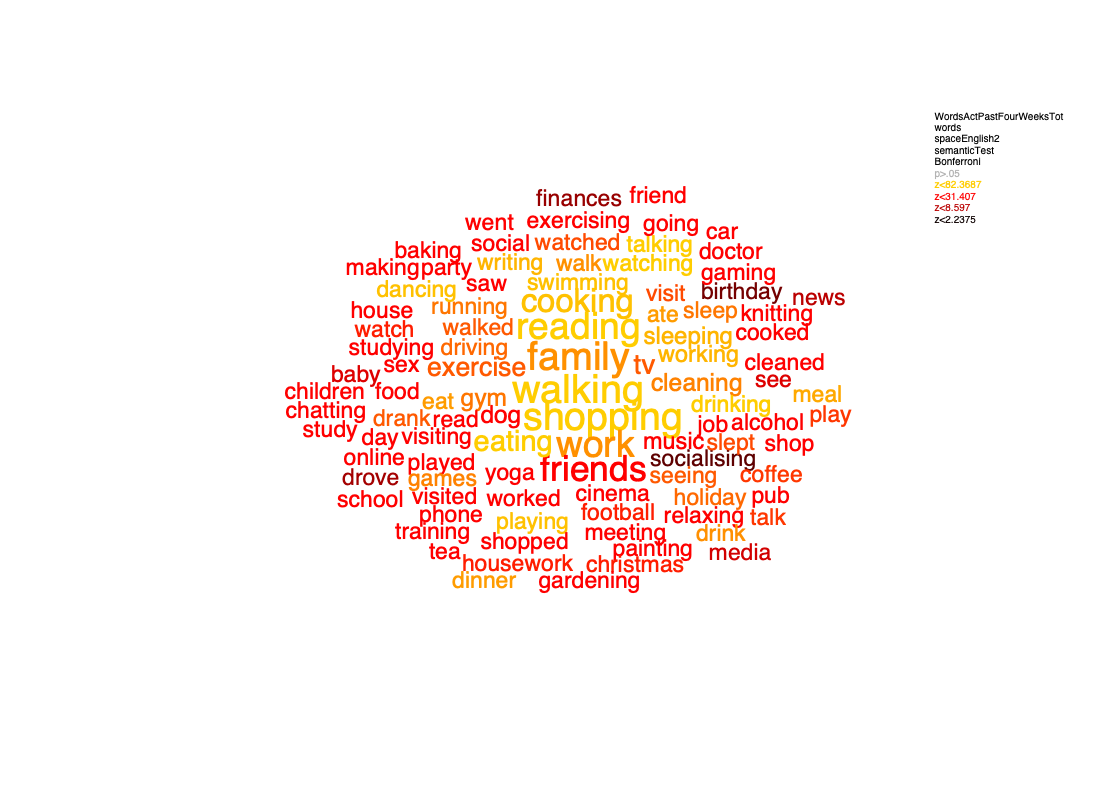


*Figure S1.3*. Activities having the most impact on SWB in the past four weeks. Semantic t-tests were computed where the words were compared to the frequency of how the words are generally used in the corpus *English 2*. Font size indicates frequency and colour indicates level of Z-transformed *t*-value. Z-values ranged from 82 to 2.3, darker colour indicates lower Z-value. All words were significant at *α* < .05. *r* = .71.2, *t* = 41.6, *p* < .01, Cohen’s *d* = 1.3.

*Figures S1.4a-d* show activities having the most impact on SWB plotted on the four SWB scales. Relational (e.g. *friends* and *family*) and exercising (e.g. *gym* and *walking*) activities were generally associated with high SWB. On the other hand, duties (e.g. *work* and *cooking*) and passive (e.g. *reading* and *tv)* activities were associated with low SWB.

**Fig S1.4. Activities with the most impact on well-being plotted along Subjective Well-Being dimensions**

*
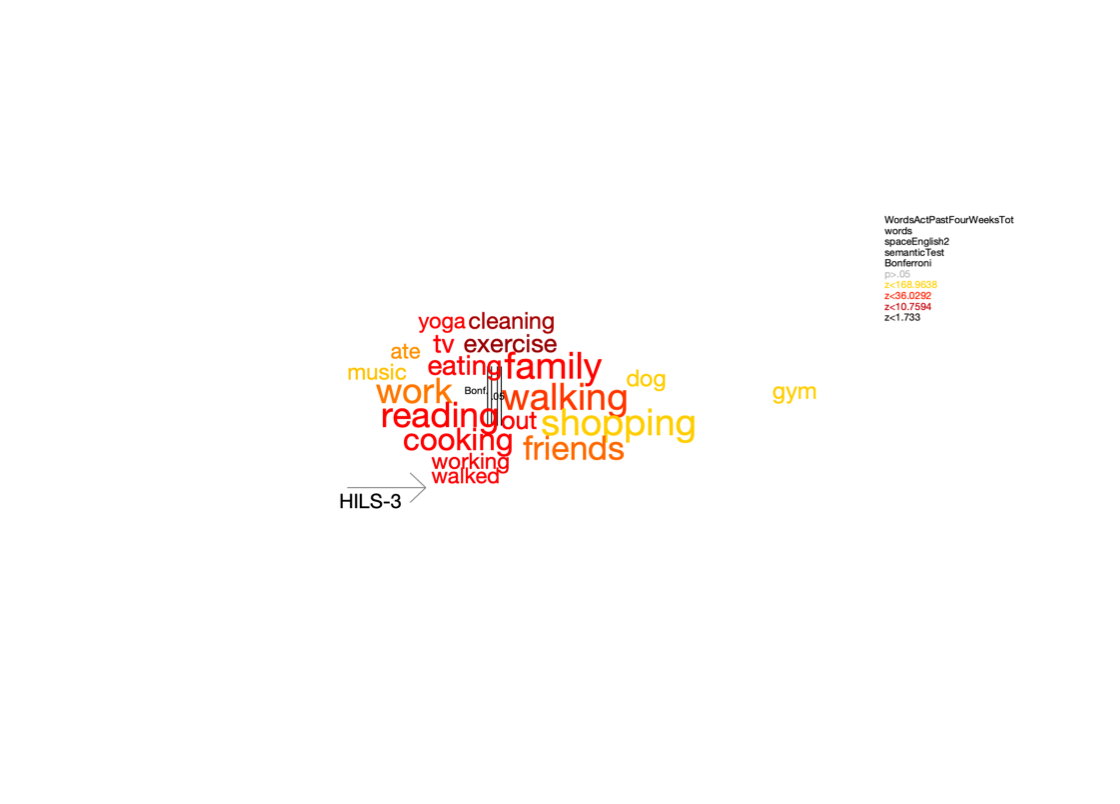

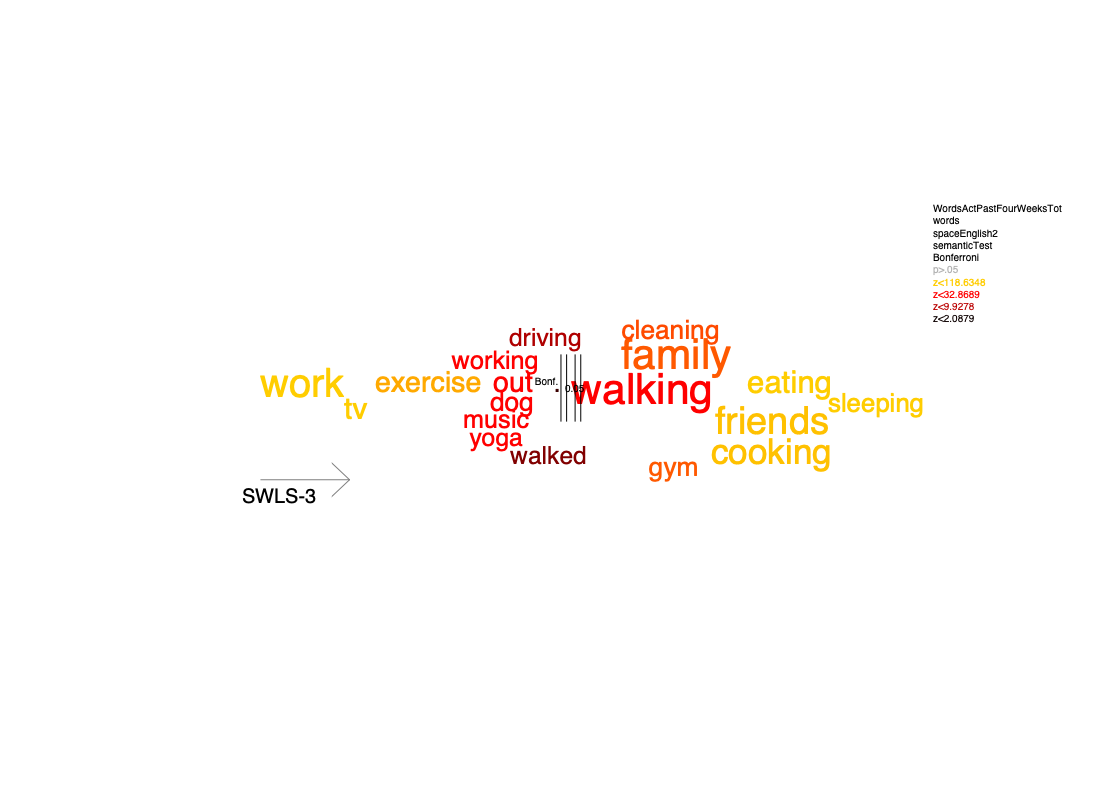
*

*S1.4a. r* = .19, *t* = 9.94, *p* < .01, *S1.4b*. *r* = .18, *t* = 9.44, *p* < .01, Cohen’s *d* = .28 Cohen’s *d* = .26

*
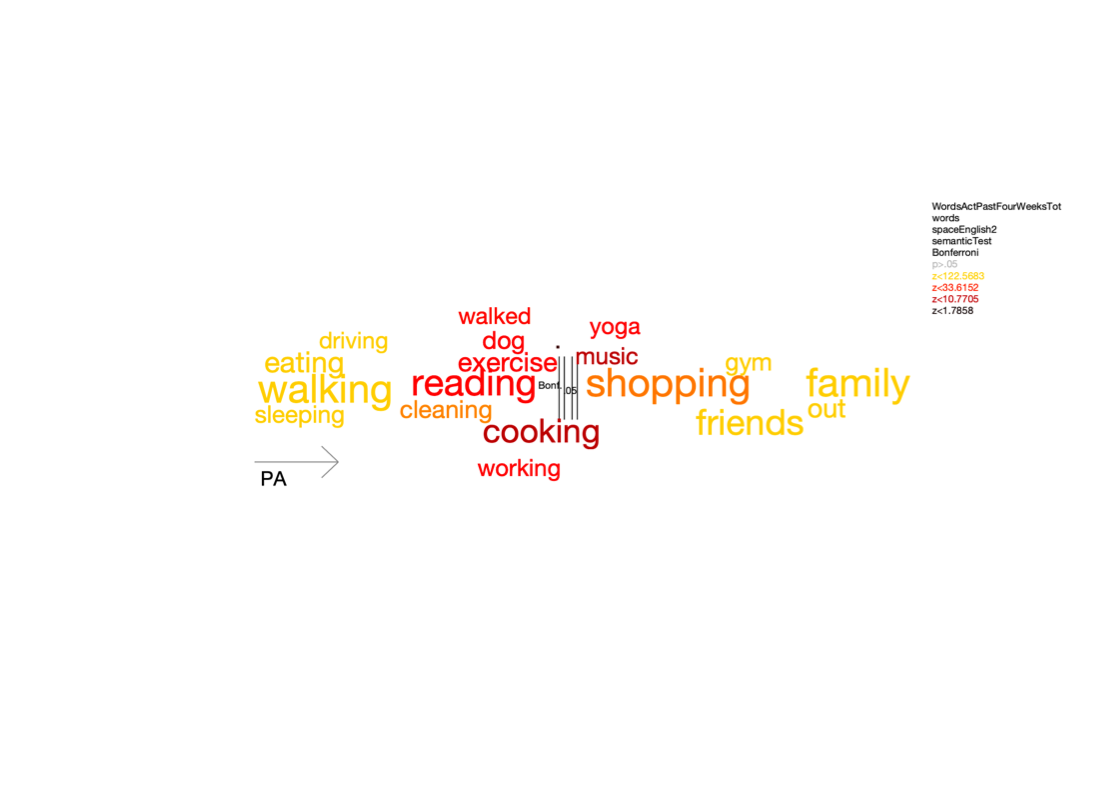

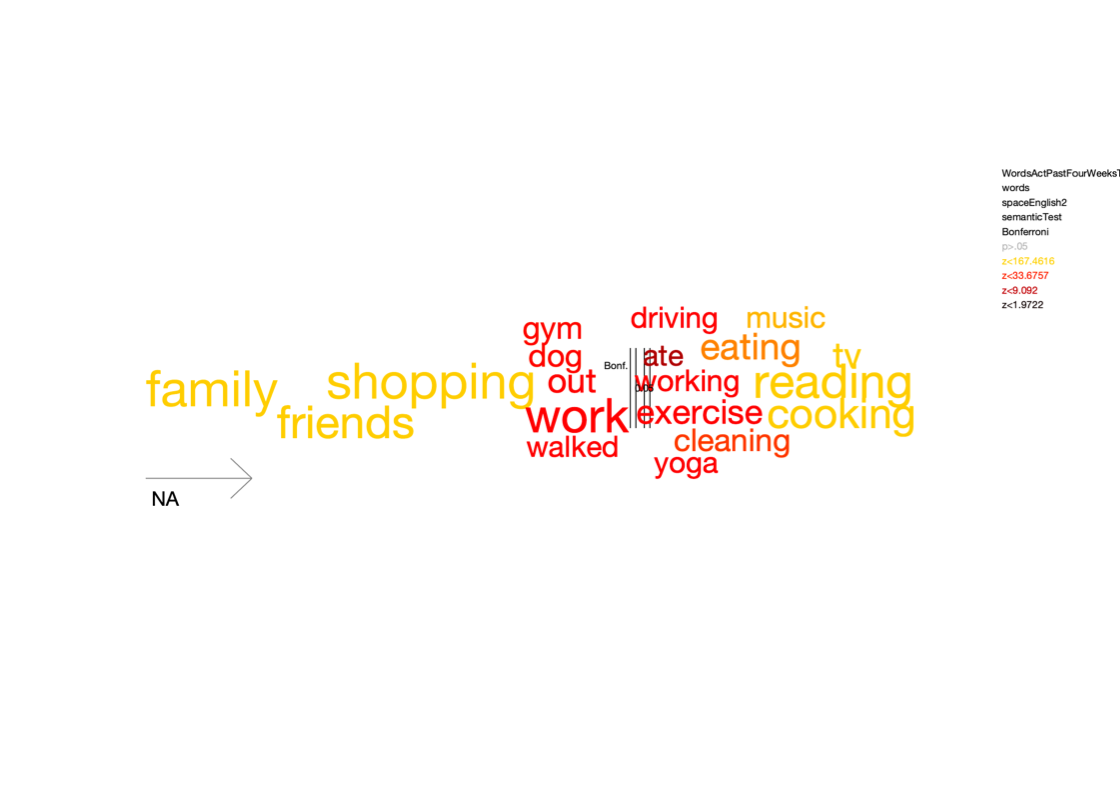
*

*S1.4c*. *r* = .18, *t* = 9.24, *p* < .01, *S1.4d*. *r* = .17, *t* = 8.6, *p* < .01,
Cohen’s *d* = .26 Cohen’s *d* = .24

*Figures S1.4a-d.* Activities having the most impact on SWB in the past four weeks plotted on the four SWB scales. Significant keywords (*p* < .05) were plotted based on semantic *t*-tests for all individual words on the semantic content of yesterday’s activities between high vs. low SWB scorers. The font size indicates word frequency and colour indicates the level of the Z-transformed *t*-values. Z-values ranged from 119 to 1.7, darker colour indicates lower Z-value. Words further away from the middle have higher Z-values. The plots show the 20 words with highest Z-scores. Plot *S1.4a*, HILS-3 = Harmony in Life Scale three item version, plot *S1.4b*, SWLS-3 = Satisfaction with Life scale three item version, plot *S1.4c*, PA = Positive Affect, plot *S1.4d*, NA = Negative Affect.

**Fig S1.5. Yesterdays’ activities along Subjective Well-Being dimensions covaried for the remaining dimensions**

*
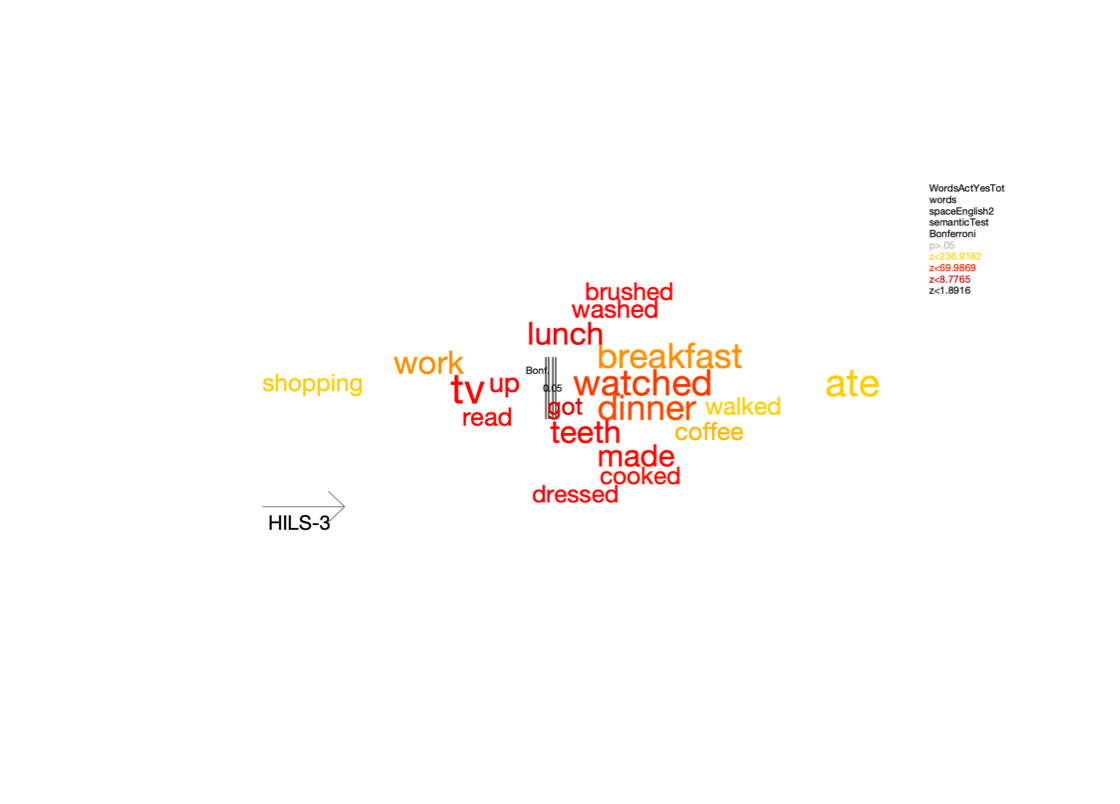

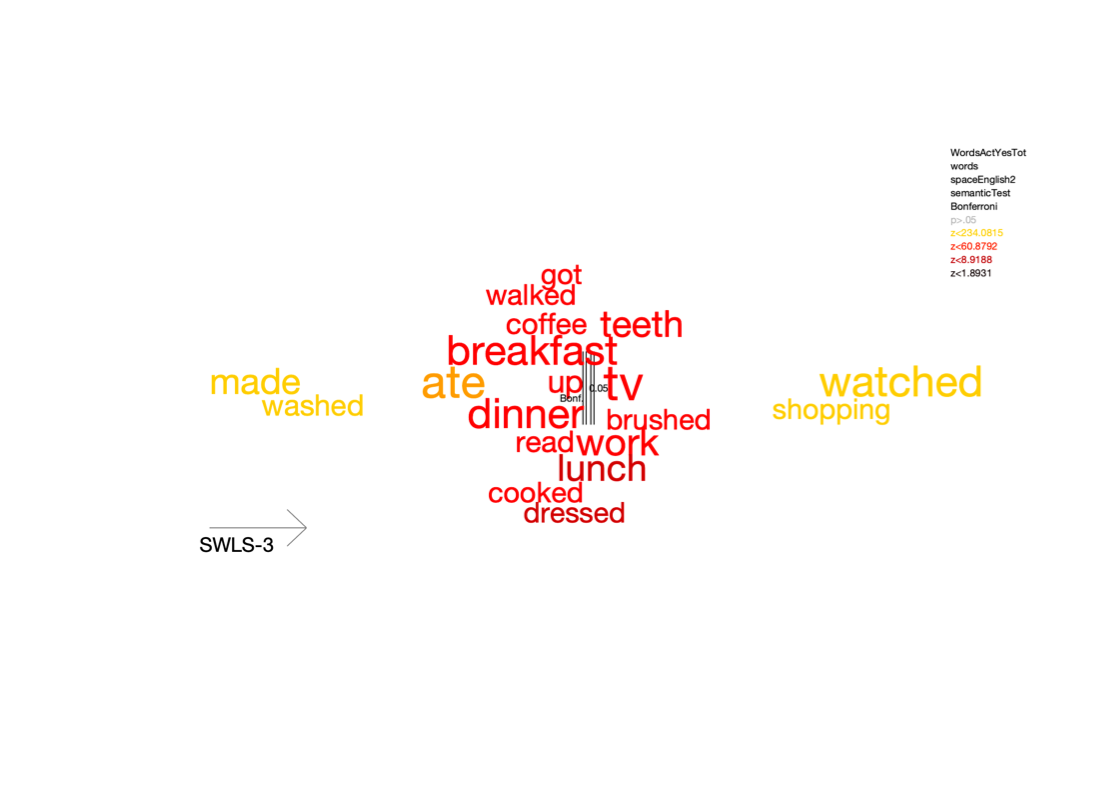
*

*S1.5a*. *r* = .15, *t* = 11.7, *p* < .01, *S1.5d*. *r* = .12, *t* = 9.8, *p* < .01,
Cohen’s *d* = .21 Cohen’s *d* = .18.

*
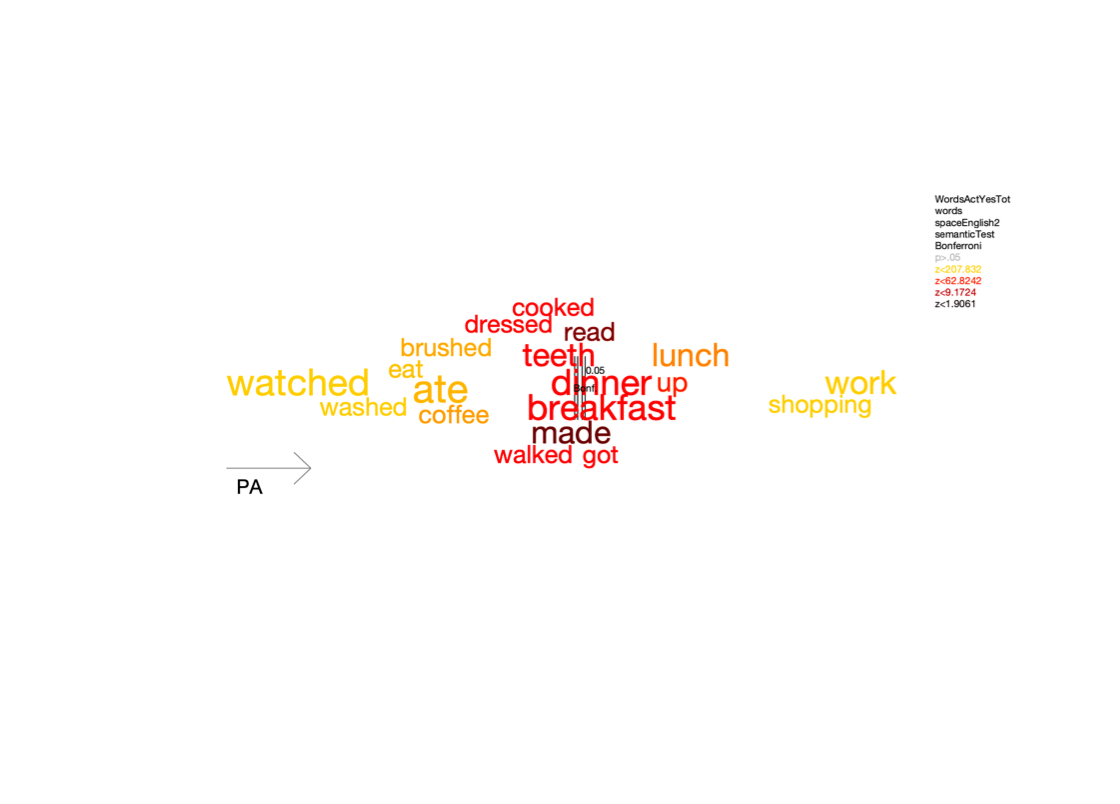

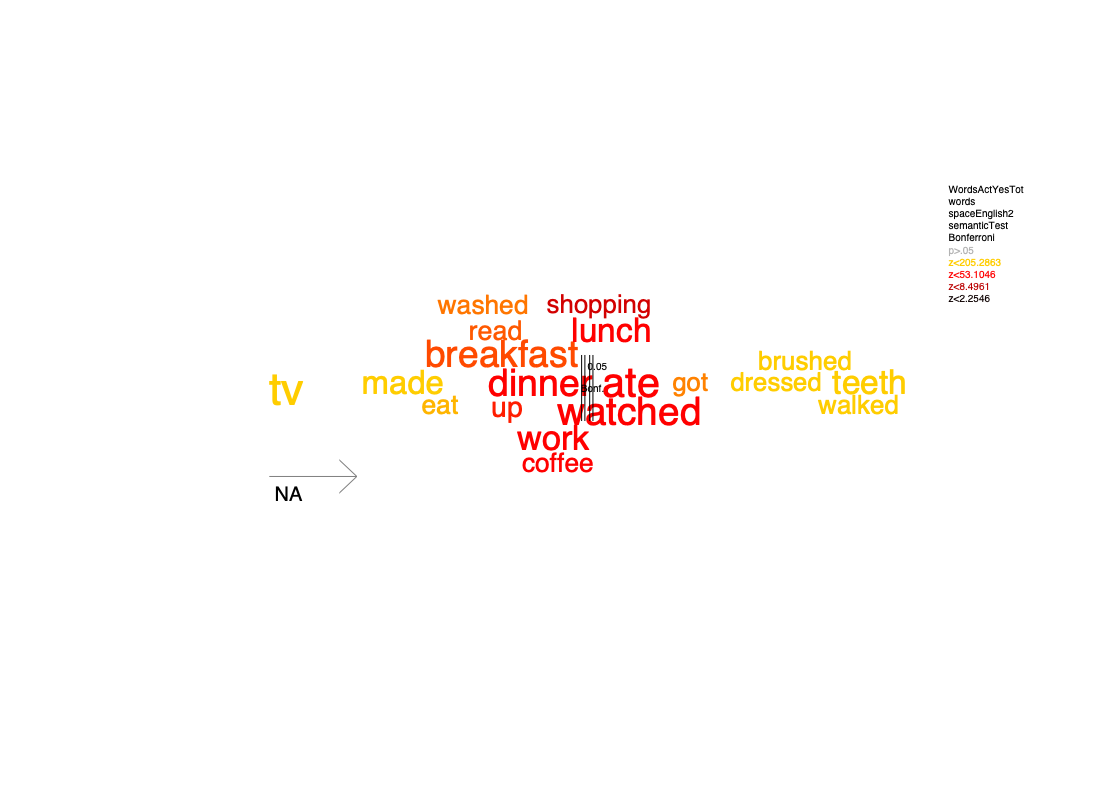
*

*S1.5c*. *r* = .15, *t* = 11.7, *p* < .01, *S1.5d*. *r* = .11, *t* = 9.0, *p* < .01,
Cohen’s *d* = .21 Cohen’s *d* = .16

*Figures S1.5a-d*. Yesterday’s activities plotted on the four SWB scales, covaried for the remaining three. Significant keywords (*p* < .05) were plotted based on semantic *t*-tests for all individual words on the semantic content of yesterday’s activities between high vs. low SWB scorers. The font size indicates frequency and colour the level of the z-transformed t-values. Z-values ranged from 236 to 1.9, darker colour indicates lower Z-value. Words farther away from the middle have higher Z-values. The plots show the 20 words with highest Z-scores. Plot *S1.5a*, HILS-3 = Harmony in Life Scale three item version, plot *S1.5b*, SWLS-3 = Satisfaction with Life scale three item version, plot *S1.5c*, PA = Positive Affect, plot *S1.5d*, NA = Negative Affect.

**Covaried word plots yesterday.** Compared to figures *S1.*2a-d, in figures *S1.*5a-d, activities are plotted along the SWB scales but also covaried with the other three SWB scales. All scales yield similar results except the *SWLS-3,* which taps more into passive activities (“watched”, “tv”, “work” and “shopping”), which were mainly in the negative half of the non-covaried plots.

**Fig S1.6. Activities with the most impact on well-being along Subjective Well-Being dimensions covaried for the remaining dimensions**


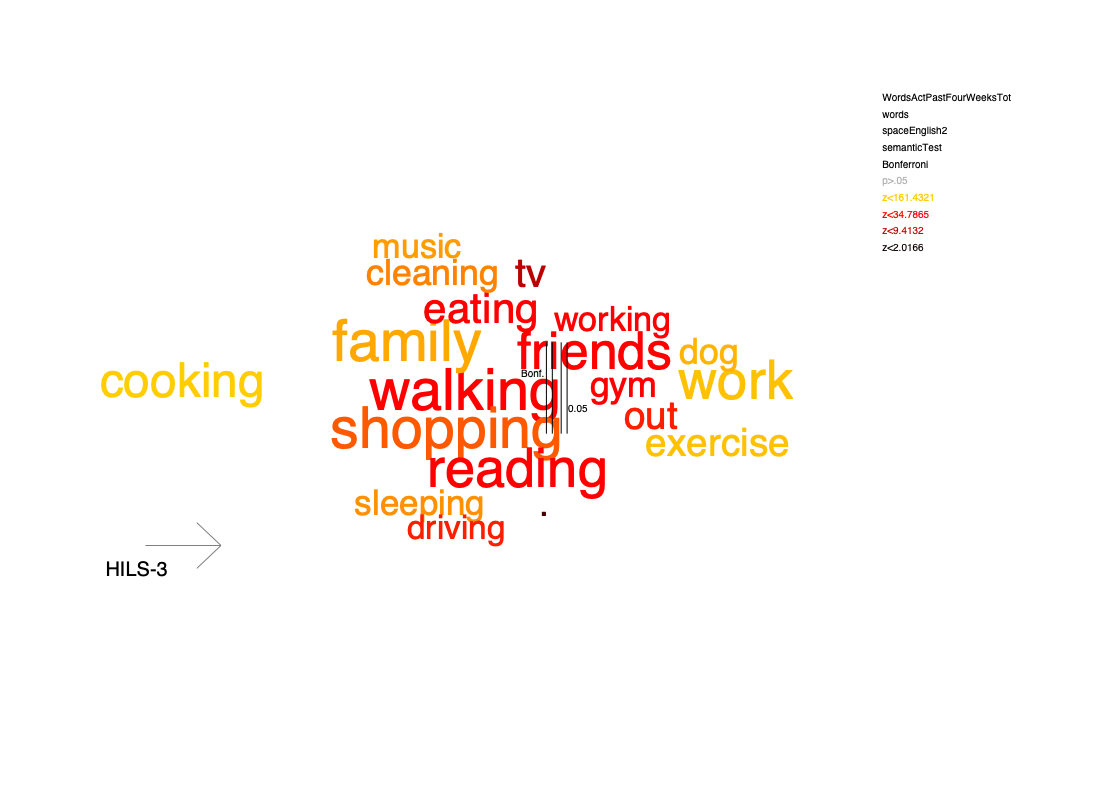

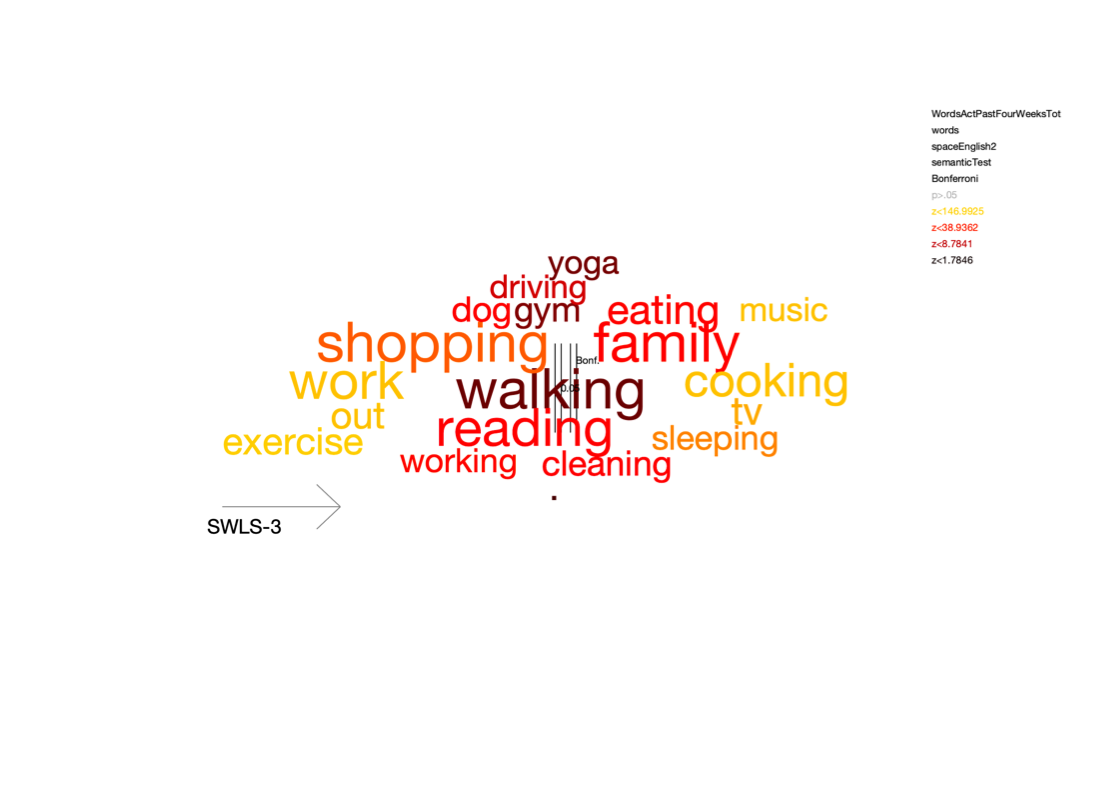


*S1.6c*. *r* = .16, *t* = 8.3, *p* < .01, *S1.6d*. *r* = .18, *t* = 9.3, *p* < .01,
Cohen’s *d* = .23 Cohen’s *d* = .26

*
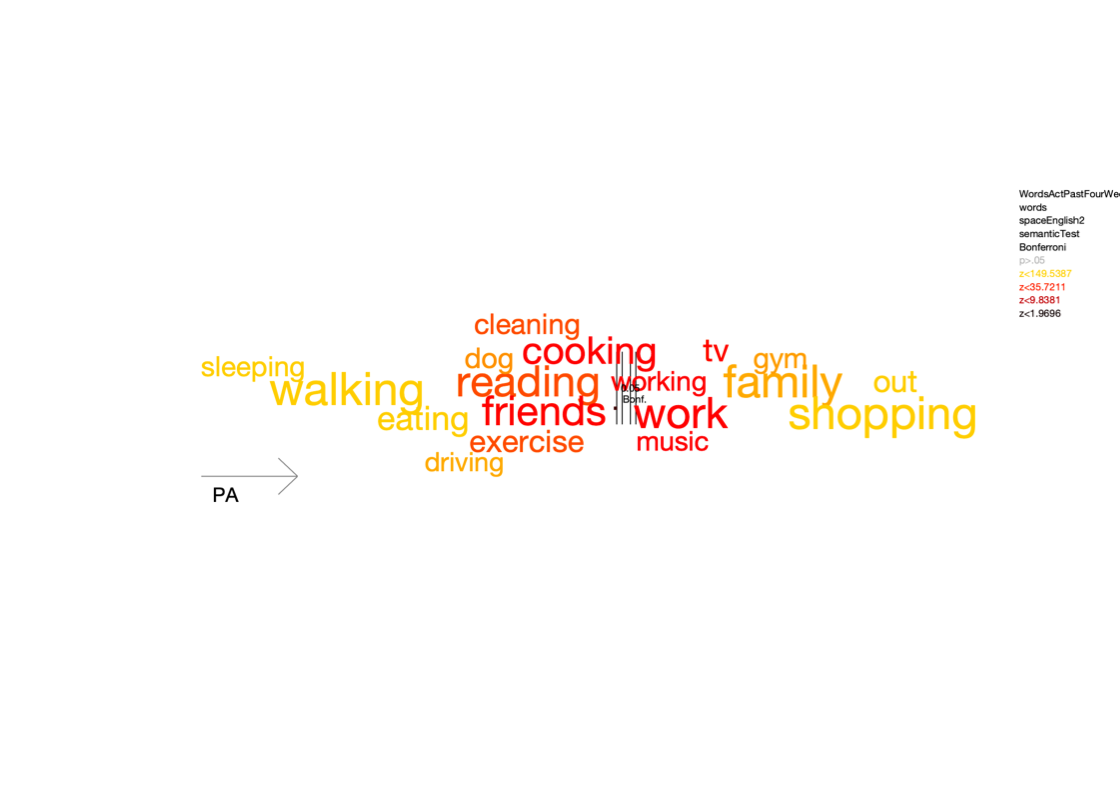

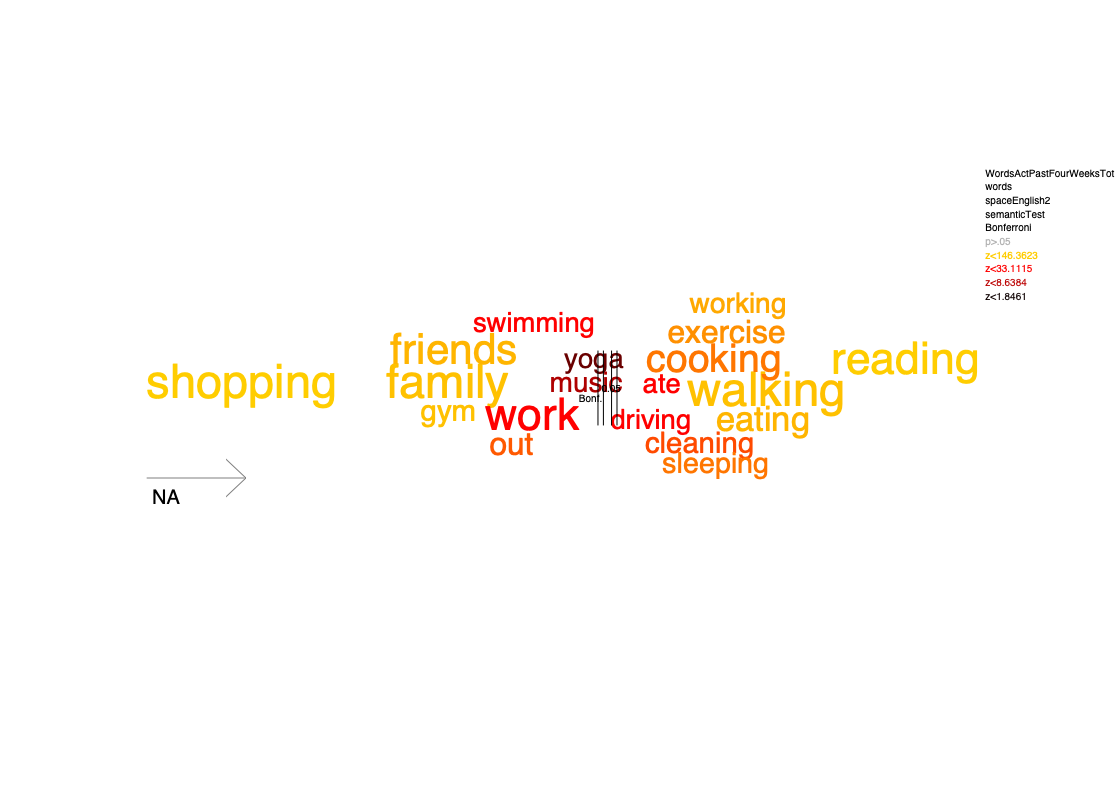
*

*S1.4c*. *r* = .19, *t* = 10.0, *p* < .01, *S1.6d*. *r* = .16, *t* = 8.0, *p* < .01,
Cohen’s *d* = .28 Cohen’s *d* = .22

*Figures S1.6a-d.* Activities having the most impact on SWB in the past four weeks plotted on the four SWB scales, covaried for the remaining three scales. Significant keywords (*p* < .05) were plotted based on semantic *t*-tests for all individual words on the semantic content of yesterday's activities between high vs. low SWB scorers. The font size indicates word frequency and colour indicates the level of the Z-transformed *t*-values. Z-values ranged from 168 to 1.8, darker colour indicates lower Z-value. Words further away from the middle have higher Z-values. The plots show the 20 words with highest Z-scores. Plot *S1.6a*, HILS-3 = Harmony in Life Scale three item version, plot *S1.6b*, SWLS-3 = Satisfaction with Life scale three item version, plot *S1.6c*, PA = Positive Affect, plot *S1.6d*, NA = Negative Affect

**Covaried word plots for activities with most impact on well-being the past four weeks.** Compared to figures *S1.*4a-d, in figures *S1.*6a-d, activities are plotted along the SWB scales but also covaried with the other three SWB scales. “Friends” seems unique for high *HILS-3* and low *NA,* whereas *shopping* seems to be more unique for low NA and high PA, and even negative for the *SWLS-3* and the *HILS-3. Reading* seems to be negative for all SWB scales even after the covaried analysis.

**Fig S1.7. Yesterdays’ activities plotted along arousal and valence**


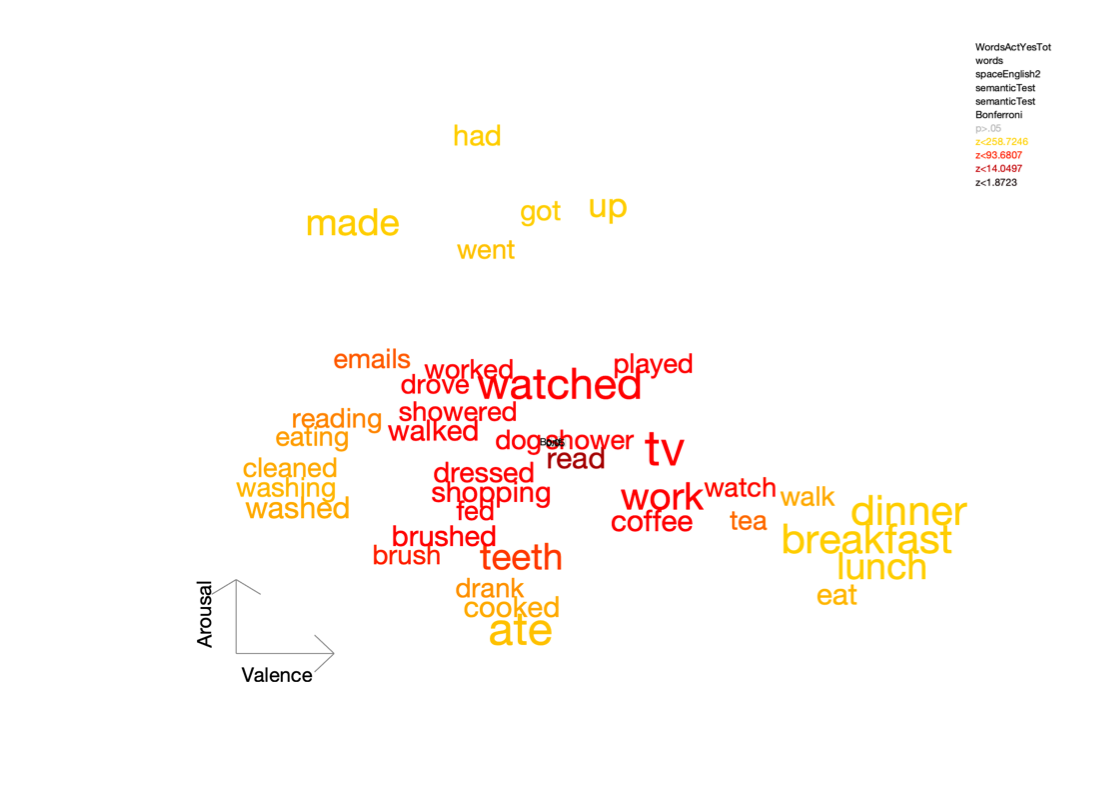


*Figure S1.7*. Yesterday’s activities plotted on valence and arousal. On the x-axis, words are plotted according to the predicted valence of the semantic representation of each participants all activities listed. On the y-axis, words are plotted according to predicted arousal of the semantic representation of each participant's all activities listed. Font size indicates frequency and colour indicates level of Z-transformed *t*-value. Z-values ranged from 256 to 1.9, darker colour indicates lower Z-value. All words were significant at *α* < .05. *r* = .27, *t* = 22.4, *p* < .01, Cohen’s *d* = .39.

**Fig S1.8. Activities with the most impact on well-being plotted along arousal and valence**

.
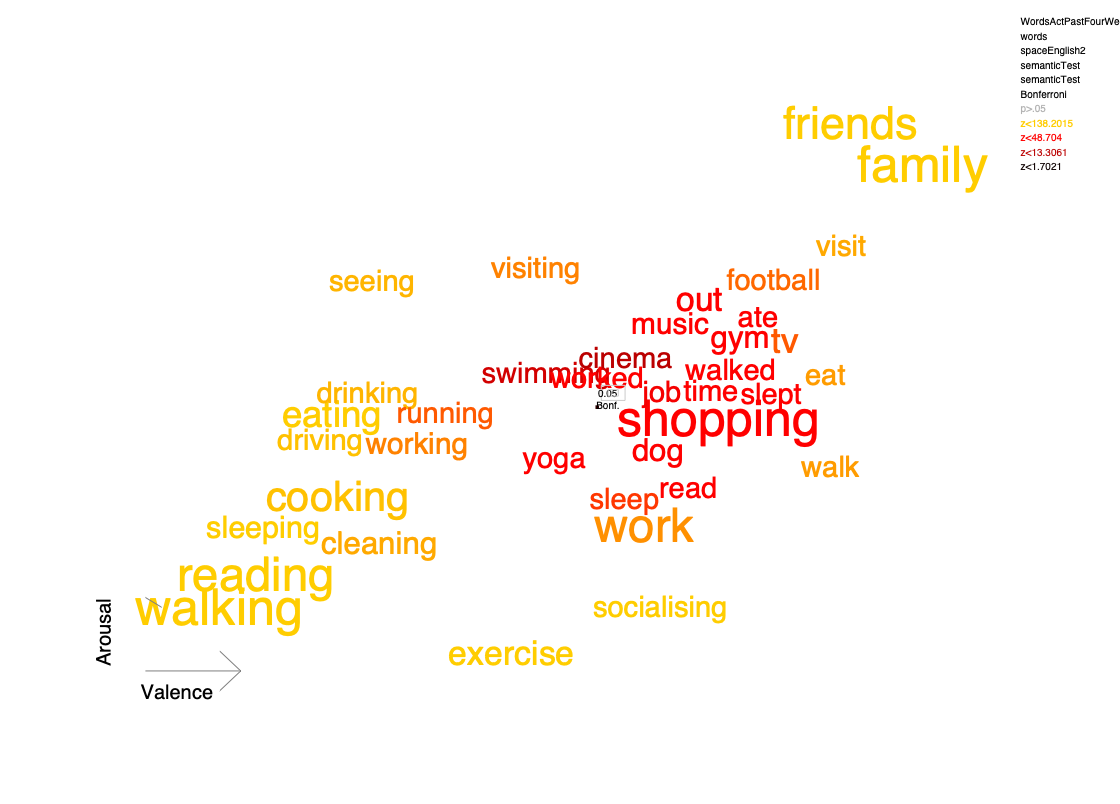


*Figure S1.8*. Activities for the past four weeks plotted on valence and arousal. On the x-axis, words are plotted according to the predicted valence of the semantic representation of each participant's all activities listed. On the y-axis, words are plotted according to the predicted arousal of the semantic representation of each participant's all activities listed. Font size indicates frequency and colour indicates level of Z-transformed *t*-value. Z-values ranged from 129 to 2, darker colour indicates lower Z-value. Font size indicates frequency and colour indicates level of Z-transformed *t*-value. Z-values ranged from 138 to 1.7, darker colour indicates lower Z-value. All words were significant at *α* < .05. *r* = .35, *t* = 18.9, *p* < .01, Cohen’s *d* = .53.

**Arousal and valence.** Activities were plotted along arousal and valence level. For yesterday’s activities (*Figure S1.7*) participants reporting activities with a high score of predicted valence and low score of predicted arousal often wrote “dinner”*,* “breakfast” and “lunch”. Participants reporting activities with a low score of predicted valence and high score of predicted arousal often wrote “made”*,* “had” and “went”. For activities having the most impact on well-being the past four weeks (*Figure S1.8*), participants reporting activities with a high score of predicted valence and arousal often wrote “friends” and “family”. Participants reporting activities with a low score of predicted valence and arousal often wrote “reading” and “walking”.

**Discussion**

**Plots.** Even though activities cannot predict SWB, the plots reveal words and activities that are associated with high and low SWB. From analyses of the word plots, the clearest pattern is that high SWB is associated mainly with food-related activities, and low SWB is associated with duties and passive activities. For activities having the most impact on well-being during the past four weeks, high SWB is related to social and active activities (such as family and friends or gym and outdoors activities), whereas low SWB is associated with duties and passive activities (such as watching TV, cooking, working and cleaning). This is consistent with the broaden-and-build theory (Fredrickson, 2001) and the Fredrickson and Branigan’s (2005) finding that people induced in a good mood have more urges to be social, exercise and be outdoors, along with many previous findings regarding activities that are associated with high SWB (Okun et al., 1984; Diener & Seligman, 2002). The findings from the word plot analyses should, however, be interpreted with caution, as the trained statistical models based on activities could not predict SWB. The activity categories mentioned (e.g “social” and “active”) are our own definitions, based on patterns found in the word plots.

**Supplementary Material Study 2**

**Table S2.1. Descriptive statistics for the numerical variables.**

**Variables Mean *SD* Skew Kurtosis**

**HILS-3**  13.34 4.77 -.61 -.69

**SWLS-3** 13.23 4,62 -.51 -.80

**PA scale**  32.85 8.35 -.41 -.39

**NA scale**  23.00 8.87 .57 -.52

**SWB** 0.00 2.5 -.48 -.53

**Valence HIL** 6.05 1.79 -.38 -.52

**Valence SWL** 6.05 1.83 -0.41 -0.77

**Valence Activities** 6.3 1.33 -0.3 -0.37

**reflecting SWB**

**Bipolar SSS HILS-3** -.01 .04 -.3 -.8

**Bipolar SSS SWLS-3** .03 .04 -.44 -.57

**Unipolar SSS HILS-3** .47 .04 -.15 -.62

**Unipolar SSS SWLS-3** .49 .04 -.82 1.5

*Note*. N = 293. HILS-3 = Harmony in Life Scale three item version, SWLS-3 = Satisfaction with Life scale three item version, PA = Positive Affect, NA = Negative Affect, SWB = Subjective Well-Being composite score Valence = predicted valence, SSS = Semantic Similarity Score.

**Table S2.2. Pearson correlations between SWB scales scores.**

**Variables 1 2 3 4 5 6 7 8 9 10 11**

**1. HILS-3**  -

**2. SWLS-3**  .83 -

**3. PA scale** .66 .62 -

**4. NA scale** -.62 -.53 -.44 -

**5. SWB** .88 .83 .83 -.81 -

**6**. **Valence HIL** .59 .50 .42 -.44 .56 -

**7. Valence SWL** .61 .60 .45 -.51 .63 .53 -

**8. Bipolar SSS HILS-3** .59 .50 .39 -.42 .54 .87 .48 -

**9. Bipolar SSS SWLS-3** .57 .60 .45 -.41 .58 .49 .83 .50 -

**10. Unipolar SSS HILS-3** .39 .34 .21 -.26 .33 .64 .40 .64 .40 -

**11. Unipolar SSS SWLS-3** .31 .33 .29 -.25 .35 .37 .56 .33 .49 .34 **-**

*Note*. *N* = 293. All *p* < .001 (2-tailed). HILS-3 = Harmony in Life Scale three item version, SWLS-3 = Satisfaction with Life Scale three item version, PA = Positive Affect, NA = Negative Affect, SWB = Subjective Well-Being composite score, Valence = predicted valence, SSS = Semantic Similarity Score.

**Table S2.3. Increasing and decreasing activities with highest/lowest dot product projections**

**Words n *dot.x***  **Words n dot.x**

**Activities increasing SWB Activities decreasing SWB**

Swimming 15 6.22 Unhealthily 3 -4.52

Badminton 2 4.91 Much 104 -4.28

Walking 103 4.84 Unhealthy 29 -3.93

Football 13 4.69 Too 138 -3.78

Tennis 3 4.56 Not 78 -3.71

Dancing 11 4.55 Enough 48 -3.55

Dance 5 4.07 Lot 8 -3.34

Exercise 59 4.06 Very 2 -3.31

Comedy 2 3.99 Than 2 -3.24

Violin 2 3.97 Can’t 2 -3.23

Singing 8 3.94 All 11 -3.17

Weightlifting 2 3.92 Lots 9 -3.08

Hockey 3 3.89 Badly 3 -3.00

Running 42 3.62 Plenty 3 -2.96

Dogs 4 3.61 Don’t 12 -2.91

Yoga 44 3.57 Bit 2 -2.84

*Note*. N_participants_ = 293.

**Unipolar SSS and Activities Reflecting SWB.**

The Unipolar SSS for SWL, had a kurtosis of 1.5 and skew of .82. However, the Leptokurtic Unipolar SSS for SWL correlated weakly to moderately with the other SWB measures which were not related to SWL, (*r* ranging from .25 to .39 including both Pearson and Spearman correlations). The relation between the Activities reflecting SWB and the Unipolar SSS for HIL and SWL are shown in *Table S2.1*.

**Table S2.4. Relation between Activities reflecting SWB and various SWB measures.**

**Activities reflecting SWB**

**Variables Training^1^ Valence^2^ BSSS**

**Unipolar SSS for HIL** .05 .10 .03

**Unipolar SSS for SWL** .07 .11 .14*

*Note*. N = 293 * *p < .05;* ** *p* < .01 (2-tailed). Valence = predicted valence of text responses, SSS = semantic similarity score, HIL = Harmony in Life, SWL = Satisfaction with Life Scale, ^1^ = Activities reflecting SWB was trained to predict the scales, ^2^ = Predicted valence of the Activities reflecting SWB

**Word plots.**

A *centrality plot* is based on a centrality point, which is an aggregated word embedding of all text data selected for the plot; and then individual words are plotted in relation to this centrality point using SSS (Kjell et al., 2021).

**Figure S2.1. Semantic Centrality Plot of Activities Reflecting SWB**


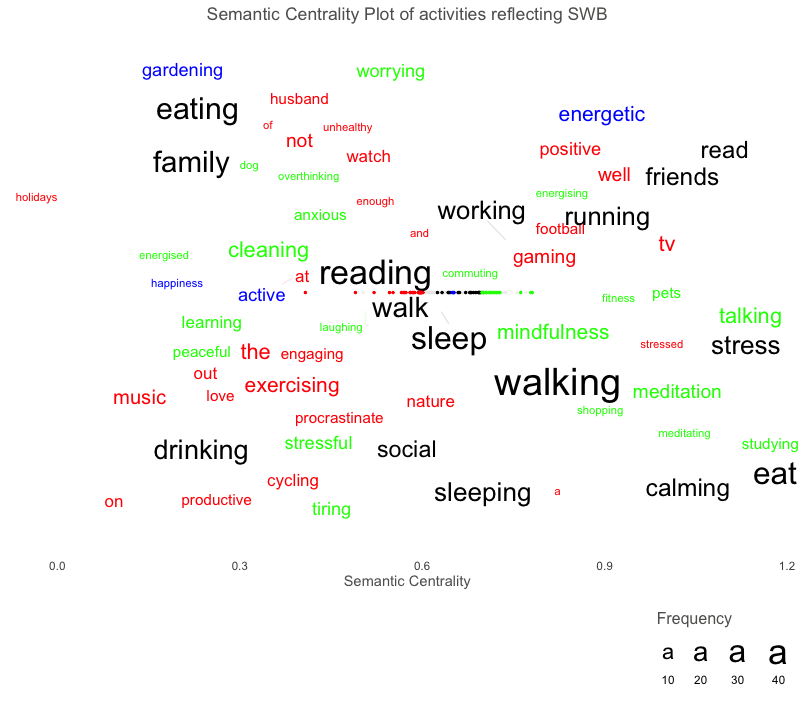


*Note:* Semantic centrality plot for Activities reflecting SWB. A *centrality plot* is based on a centrality point, which is an aggregated word embedding of all text data selected for the plot; and then individual words are plotted in relation to this centrality point using SSS [(Kjell et al., 2021)](https://www.zotero.org/google-docs/?kAO4zK). The words appearing a minimum of 3 times in the question have been compared to the aggregated word embedding of all the responses. Green words are closest to the aggregated word embedding, red words are furthest away from the aggregated word embedding and blue words are words neither close nor far away from the aggregated word embedding. The black words represent the most frequent words. Word size among all words represents frequency.

**Fig S2.2. Activities reflecting well-being for low vs high Subjective Well-Being**

***
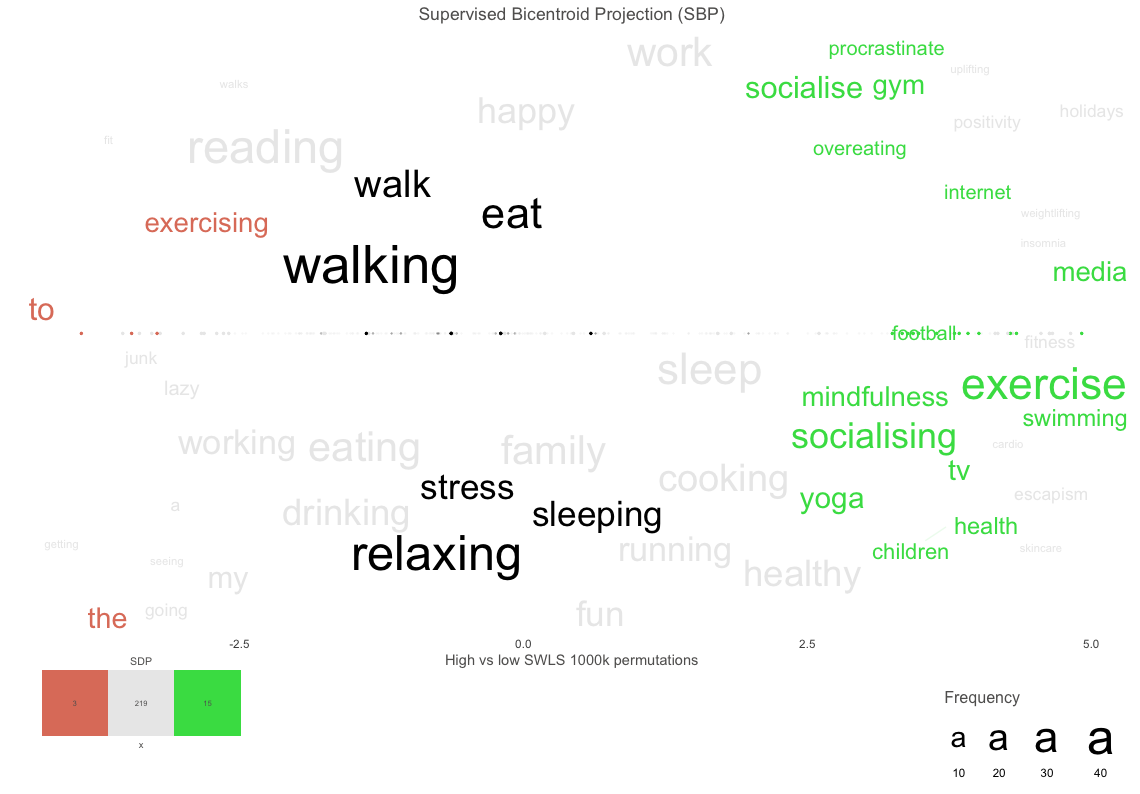
***

*Note:* Supervised dimension projection plot for activities reflecting SWB and SWB composite on the x-axis. Words significantly belonging to high SWB are plotted on the right side in green. Words significantly related to low SWB are plotted on the left side in red. Black words in the middle are frequent words not significantly belonging to any of the groups. Grey words significantly belong to the activity category of its position but occur more frequently in the other category. Word size represents frequency. The position on the dimension projection represents the dot product score. The words appearing a minimum of 5 times in the questions combined have been significance tested against a permuted null distribution, *N_permutations_*=10,000.

**References**

Bradley, M.M., & Lang, P.J. (1999). Affective norms for English words (ANEW): Instruction

manual and affective ratings. *Technical Report C-1.* The Center for Research in Psychophysiology, University of Florida. DOI: 10.3758/BF03192999

Diener, E., & Seligman, M. E. P. (2002). Very happy people. *Psychological Science*, 13(1), pp.

81-84. DOI: 10.1111/1467-9280.00415

Etkin, J., & Mogilner, C. (2016). Does variety among activities increase happiness? *Journal of*

*Consumer Research,* 43(2), pp. 210–229. DOI: [10.1093/jcr/ucw021](https://doi.org/10.1093/jcr/ucw021)

Fredrickson, B. L., & Branigan, C. (2005). Positive emotions broaden the scope of attention

and thought-action repertoires. *Cognition and Emotion*, 19(3)*,* pp. 313-332. DOI: 10.1080/02699930441000238

Fredrickson, B. L. (2001). The role of positive emotions in positive psychology: The broaden-

and-build theory of positive emotions. *American Psychologist*, 56, pp. 218-226. DOI: 10.1037//0003-066X.56.3.218

[Kjell, O., Giorgi, S., & Schwartz, H. A. (2021). *Text: An R-package for Analyzing and Visualizing Human Language Using Natural Language Processing and Deep Learning*. PsyArXiv. https://doi.org/10.31234/osf.io/293kt](https://www.zotero.org/google-docs/?FYFAyC)

Lyubomirsky, S., & Layous, K. (2013). How do simple positive activities increase well-being?

*Current Directions in Psychological Science*, 22(1), pp. 57-62. DOI: 10.1177/0963721412469809

Lawton, M. P., Winter, L., Kleban, M. H., & Ruckdeschel, K. (1999). *Journal of Aging and*

*Health*, 11(2), pp. 169–198. DOI: 10.1177/089826439901100203

Menec, V. H. (2003). The relation between everyday activities and successful aging: A 6-year

longitudinal study. *Journal of Gerontology: SOCIAL SCIENCES*, 58B(2), pp. 74–82. DOI: 10.1093/geronb/58.2.S74

Okun, M. A., Stock, W. A., Haring, M. J., & Witter, R. A. (1984). The social activity/subjective

well-being relation. *Research on Aging*, 6(1), pp. 45-65. DOI: 10.1177/0164027584006001003

Semantic Excel. Retrieved December 6, 2019, from <https://semanticexcel.com/>

Sheldon, K. M., Boehm, J. K., & Lyubomirsky, S. (2012). Variety is the spice of happiness:

The hedonic adaptation prevention (HAP) model. In I. Boniwell & S. David (Eds.), *Oxford handbook of happiness*, pp. 901–914. Oxford: Oxford University Press.

Sikström, S., Kjell, O. N. E., & Kjell, K. (2018). Semantic Excel: An Introduction to a User-

Friendly Online Software Application for Statistical Analyses of Text Data. DOI: 10.31234/osf.io/z9chp
