# Supplementary material for: Doing well-being: Self-reported activities are related to subjective well-being
Source: PLoS One. 2022 Jun 24;17(6):e0270503. doi: 10.1371/journal.pone.0270503 (PMC9232132; doi:10.1371/journal.pone.0270503)
Supplement: Attachment — Submitted filename: Response to Reviewers.docx [file pone.0270503.s002.docx]

Response to Reviewers

Dear editor and reviewers

Thank you for reviewing our manuscript and for the valuable feedback. We now believe that the manuscript has been revised according to the reviewers’ comments, including clarification of the NLP method, the presentation of the results, and the logic of the addressed research questions. In addition, we have tried our best to improve the language, and if it is not at an acceptable level at the current version, we can hopefully attain funding for a professional proofreader. Please see our detailed response to each feedback point below.

Our comments and manuscript quotes are written in **green** and manuscript changes are written in **blue**.

**Comments to the authors**

1. Is the manuscript technically sound, and do the data support the conclusions?

Reviewer #1: Partly

Reviewer #2: Partly

This has been addressed throughout our responses to the comments, particularly under comment 1 from reviewer 1 and comment 2 from reviewer 2

2. Has the statistical analysis been performed appropriately and rigorously?

Reviewer #1: I Don't Know

Reviewer #2: Yes

To reviewer 1: the NLP descriptions have been improved which might help the understanding.

3. Have the authors made all data underlying the findings in their manuscript fully available?

Reviewer #1: Yes

Reviewer #2: No

To reviewer 2: note that the data is available here <https://osf.io/8x69u/?view_only=4b72e39cc1ec4139803623ae1e67d094>

4. Is the manuscript presented in an intelligible fashion and written in standard English?

Reviewer #1: No

Reviewer #2: No

This is addressed in comment 3 from reviewer 2.

5. Review Comments to the Author

**Reviewer 1**

I gather it doesn’t count for Plos One, but actually I thought this was rather a clever idea and produced some real innovation. The paper is unusual in two ways. First, it looks at people’s past activities and then connects them to their present SWB. To my knowledge this is unusual, and maybe even a first. Second, when connecting activities to SWB it attempts to look at the “inherent” contribution to well-being that activities (however defined) might make. Good ideas both in my opinion. The overall finding is that, given these circumstances, correlations are not normally very high, maybe indicating a “well-being - activity description gap”.

Overall I found the paper hard to follow. In part I think this is because the paper is innovative & I haven’t quite got my head round it. However, I think the authors could be kinder to the reader.

We thank reviewer one for the careful consideration of the manuscript and the positive feedback. We have now made the paper clearer and kinder to the reader, e.g., by describing the NLP methods more pedagogically and by connecting the introduction to the studies more clearly.

1. The intro doesn’t really set up what is to come. So for example on p. 3 we get a lot of discussion of what SWB is and why it is a useful term and what activities are. We get discussion of passive activities (?). But we don’t get much on the role of time - how activities yesterday or in the last 4 weeks might influence how I am right now. Nor did I get properly set up for how NLP works.

Thank you for pointing this out. We have improved the introduction by increasing the set-up of the study by adding two paragraphs in which we bridge the gap between the literature and our studies. We also now clearly discuss the time aspect and illustrate that we are focusing on retrospect. We have added the following text to the introduction:

**Can individuals describe the activity – well-being relation?**

“By using Computational Language Assessments it is possible to predict individuals’ overall SWB from their self-reported (past) everyday activities. One gap within the literature is the lack of allowing individuals to describe their overall everyday activities of a given time span retrospectively and open-endedly. The studies have asked participants if they have done certain types of activities [9, 15], what they would like to do [28], or asked for everyday activities at random moments during the day for a week [8, 29]. Further, the literature has tackled the activity – well-being relation from different angles, and it seems like activities are related to SWB [6, 7, 8, 9, 10, 11, 29]. However, to our knowledge, no one has tested whether individuals can describe the relation between their past everyday activities and their overall SWB.

In our studies, we first test a broad assessment of retrospective everyday activities by letting individuals report all the activities they did yesterday (Study 1), and secondly, they are asked to report which activities they have done that impact (Study 1) or reflect (Study 2) their well-being. Both studies examine how individuals’ recall of activities relate to their self-reported subjective well-being (i.e., their subjective understanding). These two latter questions can help us understand the extent individuals understand the relationship between their everyday activities and SWB.

“How NLP works” is addressed in the third point below.

2. Bottom p. 8. The description of the SWB composite looks wrong. PANAS seems not to be in it. Check.

Thank you for pointing out this unclarity. We computed the mean of the HILS-3 and the SWLS-3 in order to represent the cognitive component, which is only the SWLS in the standard SWB composite. The affective component was treated as is typical in the literature: That is, the Cognitive component + PA - NA. We have now clarified the issue.

From:

*“Instead of the SWLS, we computed the SWB composite score using the average of the standardized scores from the SWLS-3 and the HILS-3, representing the cognitive component of SWB.”*

To:

*“*We used a similar SWB composite score, but we substituted the SWLS to the average of the standardized scores from the SWLS-3 and the HILS-3 (Positive Affect score + ((SWLS-3 + HILS-3)/2) - Negative Affect score).*”*

3. Pp 10-11. Maybe have another go at discussing NLP and word embeddings. Sorry, I don’t get it. I get the Valence idea (I think). It means that there is a list of words that have a valence. So “cat” could be good. So if the word cat is mentioned by a participant, this is good (but how good?). But there is also “training”. What happens here? Is this coming about because happy people in the study have tended to mention “cat” or have they mentioned “playing with the cat” as increasing their SWB or, well, what? I think the info may be there but I’m not getting it.

Thank you for pointing this out. We have now further clarified how the NLP methods work. We added a new paragraph to clearly explain word embeddings.

“A word embedding is a numeric representation of a word or a group of words. As such, the word embedding represents words through (many) numeric values, with the aim of capturing the latent meaning of the word(s) or text(s). The word embeddings are vectors in which the numbers can be seen as coordinates in different dimensions that describe the position of a word in a high dimensional space; and the closer two word embeddings are positioned in this space, the more similar they are in meaning. The word embeddings of individual words can be aggregated to represent several words (for more details on word embeddings, see [40]). These word embeddings are the basis for the language-based statistical analyses in this study; they are for example used to predict SWB scales score.”

We have also changed the formulations and descriptions of training, valence, and the other analysis to make it easier to follow. We hope the changes will be sufficient for the general understanding of the NLP method in the manuscript.

4. The figures are very hard to interpret. In part this is because for me Figure 2 came before Figure 1, but also the axes are not well-labelled. Indeed I’m not sure the y-axis has any significance at all. I think that in Figure 1 having, say, “walking” on the LHS and “walked” on the RHS is actually in line with the theorising but again I’m not sure. (In Figure 2 this doesn’t & shouldn’t happen.)

Thank you for pointing out the unclarity. We have now added the following information to the figure notes:

“The words are separated on the y-axis to be better seen but the y-axis does not represent any information”. Regarding the x-axis, we added the blue words of the following sentence in the figure descriptions “The position on the dimension projection (the position on the x-axis) represents the dot product score.”

Thank you also for pointing out the unclarity on walking vs walked. The existence of the same verbs in different tenses actually yield some interesting information and we added the following paragraph to the discussion section of Study 1 pp:

*“*Fig 1 reveals that participants mainly used past tense to describe Yesterdays’ activities and present participles to describe activities in the past four weeks. The difference in verb tense makes it possible for the same word stem to appear on both sides of the plot and thus “cook(ing)ed”, “dr(iving)ove”, “walk(ing)ed” and “stud(ying)ied” can be interpreted as common activities yesterday that participants perceive have impacted their well-being during the past four weeks. However, the direction of the impact remains unclear.”

5. The first para on p. 15 raises the nasty question of what different activities mean for different people and does tend to call the logic of the research into question.

Indeed a very valid point. We test this in Study 2, which is now clarified in the end of the referred paragraph:

“...We will test this level of agreement in Study 2.”

In the General Discussion we then elaborate the findings:

“The Study 2 results demonstrate that individuals show strong agreement over which activities they relate to increasing versus decreasing SWB, in line with H1. The logistic regression model categorizes the participants' full answers with an exceptionally high accuracy and the model for individual activities as increasing vs decreasing SWB activities with a very high accuracy. The results demonstrate that individuals overall share an understanding about which activities that increase versus decrease their SWB.”.

6. P. 16. Why are “cleaning” and “work” passive? This calls the active-passive dimension into question. Maybe it needs relabelling.

Thank you for pointing this confusion out. We did not intend for “cleaning” and “work” to be seen as passive. So this has been clarified.

“...whereas activities perceived as decreasing SWB are expected to relate to passive activities, such as “TV”, and ordinary everyday activities in the form of duties, such as ~~“TV”,~~ “cleaning” and “work”...”

7. P. 19. A related issue. Why are meditation and mindfulness active? I started wondering if there was some other source of categorising activities that could be usefully brought into the argument. I don’t know - not my field.

Thank you for bringing this up. In the case of meditation and mindfulness, we refer to them as *cognitively* active, which we differentiate to *physically* active activities. We have now clarified what we mean by adding the blue text coloured:

“High SWB associates with many cognitively and/or physically *active activities*. Cognitively active in the sense that one is actively engaged and focused on the activities one does, without necessarily being physically active.”

8. Table 3. Check the r for NA under Training. Looks as though it should be negative.

Significant predictions based on training cannot have negative correlations because the correlations are between the predicted values and actual scores. We have clarified this in the NLP method section by adding the following sentence in the ***Computational Language Assessment*** paragraph:

“Note that since the correlations are based on predicted values, significant correlations should always be positive.”

9. The well-being activity description gap. Some interesting questions raised here. Would it be different with happiness or indeed with well-being rather than subjective well-being? I also wonder about the consequences of not regarding work or cleaning as an activity here.

Thank you for commenting on this. We consider these very interesting empirical questions that we have included in the Future Research section:

“Considering different SWB aspects, future research may use this computational language assessment method to examine different dimensions of well-being (such as *happiness*), by asking individuals to describe their happiness in activities and words, and explore how it affects the well-being - activity description gap. Further, it is also possible to ask individuals to report specific categories of activities, such as focusing on work and duties activities, or focusing on reporting activities carried out in their free time.”

We hope that our previous response (point 6) clarified that we ses “work” and “cleaning” as activities (which we call duties). To clarify this further, we have added the following blue words when we mention duties:

“Ordinary everyday activities, in the form of duties, that most people do…” on page 6 and “…and ordinary everyday activities, in the form of duties, such as ~~“TV”,~~ “cleaning” and “work”.” on page 22.

I wouldn’t expect all the issues to resolve in this paper. It is, as I said earlier, innovative, and it’s hard to get all this right at the outset (as conceded in pp 23-24). However, I do think that what is going on could be made clearer. Worth doing, because the paper takes an interesting and unusual approach.

Thank you for the positive feedback and helpful comments, which we believe have helped make the manuscript easier to follow.

**Reviewer 2**

This manuscript presents two online studies examining associations between everyday activities and well-being using natural language processing. The manuscript uses a unique and novel approach to the question how people expect their daily activities to relate to their own well-being. Moreover, I appreciate that the data will be made available online though I was not able to access them during this review process.

However, from my point of view, there are several major issues with this manuscript that mainly concern the relevance of some research questions and the overall comprehensibility of the manuscript:

Thank you for your careful consideration, the positive feedback, and the valuable critique we hopefully can address. The data can be found [here](https://osf.io/8x69u/?view_only=4b72e39cc1ec4139803623ae1e67d094):

(1) Self-fulfilling prophecies:

Though I do think that the questions raised in this manuscript are important and insightful – particularly those regarding SWB-increasing and SWB-decreasing activities – I cannot help but wonder whether findings associating these activities to SWB may mainly be results of a self-fulfilling prophecy. Asking participants to report which of their activities in the past week(s) increased of decreased their well-being and then immediately asking participants to report said well-being may have results in a response bias in the latter questions. Thus, a more elaborate research design may be necessary.

Thank you for bringing up the important point. We believe that this is particularly important when wanting to draw conclusions about actual activities and self-reported SWB – but here we are interested in how well individuals understand this link on a subjective level. We see this as an important step before applying more elaborate (and costly studies). Throughout the manuscript we now more clearly emphasize that we are examining the subjective understanding. We have now also added the following in the “Limitations and future studies” section:

“The research in these studies asked for self-reported activities, which cannot be concluded as objectively the activities the participants actually did. Applying more elaborate research designs, such as the experience sampling method to more reliably measure activities, can potentially yield more insights into the relationship between actual activities and SWB. That could explain how big the gap is between the strength of the relationship between, on the one hand, objective activities and SWB, and on the other hand, individuals' subjective experience of the relationship.”

As a side note, the results might suggest that the self-fulfilling prophecy effect might not be that strong. When individuals report activities they *have done* that they perceive to have had the *most impact* on their well-being (Study 1), they are more likely to report occasional and exceptional activities that probably impact a subsequent SWB rating, but have little to do with actual SWB score (like a lottery win or a funeral). Compare the latter to reported *regular* activities that *reflect* well-being (Study 2). Here people would, if they understand the question, more often report everyday activities that reflect their actual SWB level, so that a person high on SWB would report “socialising” or “exercise” and other activities aligned with the right side of Fig 2 (activities increasing SWB) and a person low on SWB would report “overthinking” and other activities aligned with the left side of Fig 2 (activities decreasing SWB). Thus if the bias effect was strong, the correlations to said well-being might have been stronger in Study 1 than in Study 2, which was not the case.

(2) Activities that reflect people’s well-being:

Frankly, I have not understood properly why the question about activities reflecting well-being is relevant. On the one hand, I would intuitively assume that participants might find it very difficult to answer this question - I personally would not know ad hoc what to state there. On the other hand, the authors do not go into great detail in the introduction / derivation of the hypotheses as to why this question should be relevant to the field at all. This makes it seem more like a demonstration of the NLP method than a substantive research question.

Thank you for bringing the important issue up. We have now added a sentence in the start of the manuscript concerning the importance of the aim that the question address:

“This is important because it can help us understand the extent individuals understand the relationship between their everyday activities and SWB.”

We also added a derivation to the relevance of the question in the introduction in which we bridge the gap between the literature and our studies. We have added the following in the introduction:

**Can individuals describe the activity – well-being relation?**

“By using Computational Language Assessments it is possible to predict individuals’ overall SWB from their self-reported (past) everyday activities…

…Further, the literature has tackled the activity – well-being relation from different angles, and it seems like activities are related to SWB [6, 7, 8, 9, 10, 11, 29]. However, to our knowledge, no one has tested whether individuals can describe the relation between their past everyday activities and their overall SWB.

In our studies, we first test a broad assessment of retrospective everyday activities by letting individuals report all the activities they did yesterday (Study 1), and secondly, they are asked to report which activities they have done that impact (Study 1) or reflect (Study 2) their well-being. Both studies examine how individuals’ recall of activities relate to their self-reported subjective well-being (i.e., their subjective understanding). These two latter questions can help us understand the extent individuals understand the relationship between their everyday activities and SWB.

We also added two sentences regarding why we changed the assessment from activities with the most impact on well-being the past four weeks to activities reflecting well-being in the intro of Study 2:

“The assessment of individuals’ understanding of how their everyday activities link to their SWB is slightly different from Study 1 in two ways: first, we ask for *regular* activities instead of activities during *the past four weeks* in order to resemble everyday activities. Second, we ask about activities *reflecting* SWB instead of *impacting* SWB. The word “impact” implies a change in SWB, while “reflecting” implies an assessment of the current level.”

We hope that the added material brings clarity to the raised issue.

(3) Overall language:

Throughout the manuscript there are many grammatical errors and unusual phrasings that often made it difficult for me (as a non-native speaker) to follow the argumentation. I would therefore highly recommend the authors to revise the manuscript carefully for linguistic correctness - it is okay if the English is not perfect, but this should not affect the comprehensibility.

Thank you for commenting on this. We have paid more attention to improving the language. If this is not sufficient, we might be able to get funding for a professional proofreader.

(4) Presentation of methods and results:

As someone who is not at all familiar with the details of natural language processing, I sometimes had a hard time following the methods and results, or more precisely what they meant with respect to the research questions. Particularly in the results sections, the authors often only describe statistical results but do not explain what these mean with respect to the hypotheses or research questions. I would therefore appreciate it if the authors would revise the results section, again specifically making sure that the results are described in a generally understandable way and in relation to the research questions

Thank you for highlighting this. We have now clarified throughout the result sections what the presented analysis means for the aims of the research. For example in the beginning of the section labeled **Activities increasing and decreasing SWB plotted** we have added:

“To answer which everyday activities individuals relate to well-being, activities increasing and decreasing SWB are plotted in Fig 2 in a supervised dimension projection plot.”

And in the beginning of the section **Activities predict SWB** we have added:

“The next analysis examines whether individuals can describe how their everyday activities relate to their SWB. This is done by predicting rated SWB from the everyday activities individuals perceive to reflect their SWB.”

For the NLP analysis, we have clarified the NLP section, see comment 3 to reviewer 1.

In summary, I think this manuscript uses an intriguing method and asks some very interesting questions - but also some questions whose usefulness to the field I could not really comprehend. In my opinion, the manuscript would need to be fundamentally revised both in terms of language and in terms of the research questions and their relevance to the literature before I could recommend

Thank you for your valuable feedback. We sincerely appreciate it and strongly believe that the quality of the paper has improved thanks to it.
