# Supplementary material for: Doing well-being: Self-reported activities are related to subjective well-being
Source: PLoS One. 2022 Jun 24;17(6):e0270503. doi: 10.1371/journal.pone.0270503 (PMC9232132; doi:10.1371/journal.pone.0270503)
Supplement: Attachment — Submitted filename: Response to Reviewers - revision 2 .docx [file pone.0270503.s003.docx]

Response to Reviewers, second revision

Dear editor and reviewer

Thank you for reviewing our manuscript again. We now believe that the manuscript has been improved according to the mentioned requirements. There are many changes in the manuscript. That is due to professional proofreading, and thus only the language has been affected, including the reviewer's suggestion for the abstract. Further, we discovered an error in the reference list which made the references from source 36 upwards wrong, and one in-text reference was written by author name and not by reference number. This is also fixed.

We have a change in the financial disclosure. The funding is as follows:

*The authors August Nilsson and Erik Hellryd received no specific funding for this work.*

*The author Oscar Kjell received funding from the Swedish Research Council - https://www.vr.se/english.html Grant number 2019-06305.*

*The funders had no role in study design, data collection and analysis, decision to publish, or preparation of the manuscript.*

**Comments to the authors from the editor**

Please review your reference list to ensure that it is complete and correct. If you have cited papers that have been retracted, please include the rationale for doing so in the manuscript text, or remove these references and replace them with relevant current references. Any changes to the reference list should be mentioned in the rebuttal letter that accompanies your revised manuscript. If you need to cite a retracted article, indicate the article’s retracted status in the References list and also include a citation and full reference for the retraction notice.

We mistakenly wrote source 37 as a part of source 36. Thus, all sources after 36 were wrong. We have fixed this. Further, We wrote the name instead of the number for one of the sources. This is also fixed.

**Comments to the authors from the reviewer**

1. I was a reviewer (1) of the previous paper, and I thank the authors for their considered responses and changes to all my previous remarks. I think the paper is now more accessible and, given that it was always interesting and cleverly done, I have no suggestions for further major change. The paper will remain a little difficult for many readers but this reflects the innovative and unfamiliar work undertaken. I thought the English was always understandable. I do suggest below a patch for some of the abstract, which might improve this a little. It’s below and should be OK to cut & paste if the authors choose to. Abstract patch: In Study 2 (N=295) individuals showed strong agreement concerning which activities they considered to have increased or decreased SWB (AUC = .995). Words describing activities that increased SWB related to physically and cognitively active activities and social activities (“football”, “meditation”, “friends”), whereas words describing activities that decreased SWB mainly illustrated activity features related to imbalance (“too”, “much”, “enough”).

Thank you for the additional review, positive feedback, suggestion for improvement, and interesting thoughts. We added the suggestion of improving the abstract from Reviewer 1. We then sent the manuscript for professional proofreading. We submit both a version with accepted track changes and one including track changes.

2. On the reread I spent a bit of time thinking about eating (& various related words) in Figures 1 and 2. These activities don’t seem to do much for SWB but SWB probably would decrease it if you didn’t do them. Everyone (well, nearly everyone) eats daily and most people are reasonably happy so why would you notice a connection? Why would there be one when there is little variance? On a somewhat different track I wondered if the eating results in Figure 2 would be a bit more positive if you ran the study somewhere other than the UK. At least anecdotally most other cultures do regard food as a source of SWB. I don’t think you should feel obliged to include these points in this paper, but they might be worth considering in the future. Nice paper!

Interesting reflection about food. We agree, most daily activities are important for our SWB, like work and eating, but we might not notice the importance since they are ubiquitous for most people. And yes, culture certainly could have an impact on the relationship between food and SWB, something we hope to be able to investigate in the future.
