# Supplementary material for: Doing well-being: Self-reported activities are related to subjective well-being
Source: PLoS One. 2022 Jun 24;17(6):e0270503. doi: 10.1371/journal.pone.0270503 (PMC9232132; doi:10.1371/journal.pone.0270503)
Supplement: Peer review history

# Peer review: Decision Letter 0

**DOI:** 10.1371/journal.pone.0270503.r001

**Authors:** Darrell A. Worthy

10 Feb 2022

PONE-D-21-37441Doing Well-Being: 

Self-Reported Activities Are Related to Subjective Well-BeingPLOS ONE

Dear Dr. Nilsson,

Thank you for submitting your manuscript to PLOS ONE. After careful consideration, we feel that it has merit but does not fully meet PLOS ONE’s publication criteria as it currently stands. Therefore, we invite you to submit a revised version of the manuscript that addresses the points raised during the review process.

Two experts from the field reviewed your paper.  Reviewer 1 is somewhat more positive than Reviewer 2; however, both reviewers noted extensive issues with the paper that would require major revision.  If you'd like, you may submit a revision, and I will return the paper to the original reviewers.  If the reviewers still have major concerns with the paper, then I will likely reject the manuscript, but I invite you to submit a revision if you feel you can adequately address all of the reviewers' concerns.

Please submit your revised manuscript by Mar 27 2022 11:59PM. If you will need more time than this to complete your revisions, please reply to this message or contact the journal office at plosone@plos.org. When you're ready to submit your revision, log on to https://www.editorialmanager.com/pone/ and select the 'Submissions Needing Revision' folder to locate your manuscript file.

Please include the following items when submitting your revised manuscript:

- A rebuttal letter that responds to each point raised by the academic editor and reviewer(s). You should upload this letter as a separate file labeled 'Response to Reviewers'.
- A marked-up copy of your manuscript that highlights changes made to the original version. You should upload this as a separate file labeled 'Revised Manuscript with Track Changes'.
- An unmarked version of your revised paper without tracked changes. You should upload this as a separate file labeled 'Manuscript'.

If you would like to make changes to your financial disclosure, please include your updated statement in your cover letter. Guidelines for resubmitting your figure files are available below the reviewer comments at the end of this letter.

If applicable, we recommend that you deposit your laboratory protocols in protocols.io to enhance the reproducibility of your results. Protocols.io assigns your protocol its own identifier (DOI) so that it can be cited independently in the future. For instructions see: https://journals.plos.org/plosone/s/submission-guidelines#loc-laboratory-protocols. Additionally, PLOS ONE offers an option for publishing peer-reviewed Lab Protocol articles, which describe protocols hosted on protocols.io. Read more information on sharing protocols at https://plos.org/protocols?utm_medium=editorial-email&utm_source=authorletters&utm_campaign=protocols.

We look forward to receiving your revised manuscript.

Kind regards,

Darrell A. Worthy, Ph.D

Academic Editor

PLOS ONE

Journal Requirements:

When submitting your revision, we need you to address these additional requirements.

1. Please ensure that your manuscript meets PLOS ONE's style requirements, including those for file naming. The PLOS ONE style templates can be found at

https://journals.plos.org/plosone/s/file?id=wjVg/PLOSOne_formatting_sample_main_body.pdf and

https://journals.plos.org/plosone/s/file?id=ba62/PLOSOne_formatting_sample_title_authors_affiliations.pdf

2. Please include captions for your Supporting Information files at the end of your manuscript, and update any in-text citations to match accordingly. Please see our Supporting Information guidelines for more information: http://journals.plos.org/plosone/s/supporting-information.

[Note: HTML markup is below. Please do not edit.]

Reviewers' comments:

Reviewer's Responses to Questions

**Comments to the Author**

1. Is the manuscript technically sound, and do the data support the conclusions?

The manuscript must describe a technically sound piece of scientific research with data that supports the conclusions. Experiments must have been conducted rigorously, with appropriate controls, replication, and sample sizes. The conclusions must be drawn appropriately based on the data presented. 

Reviewer #1: Partly

Reviewer #2: Partly

**********

2. Has the statistical analysis been performed appropriately and rigorously? 

Reviewer #1: I Don't Know

Reviewer #2: Yes

**********

3. Have the authors made all data underlying the findings in their manuscript fully available?

The PLOS Data policy requires authors to make all data underlying the findings described in their manuscript fully available without restriction, with rare exception (please refer to the Data Availability Statement in the manuscript PDF file). The data should be provided as part of the manuscript or its supporting information, or deposited to a public repository. For example, in addition to summary statistics, the data points behind means, medians and variance measures should be available. If there are restrictions on publicly sharing data—e.g. participant privacy or use of data from a third party—those must be specified.

Reviewer #1: Yes

Reviewer #2: No

**********

4. Is the manuscript presented in an intelligible fashion and written in standard English?

PLOS ONE does not copyedit accepted manuscripts, so the language in submitted articles must be clear, correct, and unambiguous. Any typographical or grammatical errors should be corrected at revision, so please note any specific errors here.

Reviewer #1: No

Reviewer #2: No

**********

5. Review Comments to the Author

Please use the space provided to explain your answers to the questions above. You may also include additional comments for the author, including concerns about dual publication, research ethics, or publication ethics. (Please upload your review as an attachment if it exceeds 20,000 characters)

Reviewer #1: Review of PONE-D-21-37441 :Doing well-being…”

I gather it doesn’t count for Plos One, but actually I thought this was rather a clever idea and produced some real innovation. The paper is unusual in two ways. First, it looks at people’s past activities and then connects them to their present SWB. To my knowledge this is unusual, and maybe even a first. Second, when connecting activities to SWB it attempts to look at the “inherent” contribution to well-being that activities (however defined) might make. Good ideas both in my opinion. The overall finding is that, given these circumstances, correlations are not normally very high, maybe indicating a “well-being - activity description gap”.

Overall I found the paper hard to follow. In part I think this is because the paper is innovative & I haven’t quite got my head round it. However, I think the authors could be kinder to the reader. Some issues:

1. The intro doesn’t really set up what is to come. So for example on p. 3 we get a lot of discussion of what SWB is and why it is a useful term and what activities are. We get discussion of passive activities (?). But we don’t get much on the role of time - how activities yesterday or in the last 4 weeks might influence how I am right now. Nor did I get properly set up for how NLP works.

2. Bottom p. 8. The description of the SWB composite looks wrong. PANAS seems not to be in it. Check.

3. Pp 10-11. Maybe have another go at discussing NLP and word embeddings. Sorry, I don’t get it. I get the Valence idea (I think). It means that there is a list of words that have a valence. So “cat” could be good. So if the word cat is mentioned by a participant, this is good (but how good?). But there is also “training”. What happens here? Is this coming about because happy people in the study have tended to mention “cat” or have they mentioned “playing with the cat” as increasing their SWB or, well, what? I think the info may be there but I’m not getting it.

4. The figures are very hard to interpret. In part this is because for me Figure 2 came before Figure 1, but also the axes are not well-labelled. Indeed I’m not sure the y-axis has any significance at all. I think that in Figure 1 having, say, “walking” on the LHS and “walked” on the RHS is actually in line with the theorising but again I’m not sure. (In Figure 2 this doesn’t & shouldn’t happen.)

5. The first para on p. 15 raises the nasty question of what different activities mean for different people and does tend to call the logic of the research into question.

6. P. 16. Why are “cleaning” and “work” passive? This calls the active-passive dimension into question. Maybe it needs relabelling.

7. P. 19. A related issue. Why are meditation and mindfulness active? I started wondering if there was some other source of categorising activities that could be usefully brought into the argument. I don’t know - not my field.

8. Table 3. Check the r for NA under Training. Looks as though it should be negative.

9. The well-being activity description gap. Some interesting questions raised here. Would it be different with happiness or indeed with well-being rather than subjective well-being? I also wonder about the consequences of not regarding work or cleaning as an activity here.

I wouldn’t expect all the issues to resolve in this paper. It is, as I said earlier, innovative, and it’s hard to get all this right at the outset (as conceded in pp 23-24). However, I do think that what is going on could be made clearer. Worth doing, because the paper takes an interesting and unusual approach.

Reviewer #2: This manuscript presents two online studies examining associations between everyday activities and well-being using natural language processing. The manuscript uses a unique and novel approach to the question how people expect their daily activities to relate to their own well-being. Moreover, I appreciate that the data will be made available online though I was not able to access them during this review process.

However, from my point of view, there are several major issues with this manuscript that mainly concern the relevance of some research questions and the overall comprehensibility of the manuscript:

(1) Self-fulfilling prophecies:

Though I do think that the questions raised in this manuscript are important and insightful – particularly those regarding SWB-increasing and SWB-decreasing activities – I cannot help but wonder whether findings associating these activities to SWB may mainly be results of a self-fulfilling prophecy. Asking participants to report which of their activities in the past week(s) increased of decreased their well-being and then immediately asking participants to report said well-being may have results in a response bias in the latter questions. Thus, a more elaborate research design may be necessary.

(2) Activities that reflect people’s well-being:

Frankly, I have not understood properly why the question about activities reflecting well-being is relevant. On the one hand, I would intuitively assume that participants might find it very difficult to answer this question - I personally would not know ad hoc what to state there. On the other hand, the authors do not go into great detail in the introduction / derivation of the hypotheses as to why this question should be relevant to the field at all. This makes it seem more like a demonstration of the NLP method than a substantive research question.

(3) Overall language:

Throughout the manuscript there are many grammatical errors and unusual phrasings that often made it difficult for me (as a non-native speaker) to follow the argumentation. I would therefore highly recommend the authors to revise the manuscript carefully for linguistic correctness - it is okay if the English is not perfect, but this should not affect the comprehensibility.

(4) Presentation of methods and results:

As someone who is not at all familiar with the details of natural language processing, I sometimes had a hard time following the methods and results, or more precisely what they meant with respect to the research questions. Particularly in the results sections, the authors often only describe statistical results but do not explain what these mean with respect to the hypotheses or research questions. I would therefore appreciate it if the authors would revise the results section, again specifically making sure that the results are described in a generally understandable way and in relation to the research questions

In summary, I think this manuscript uses an intriguing method and asks some very interesting questions - but also some questions whose usefulness to the field I could not really comprehend. In my opinion, the manuscript would need to be fundamentally revised both in terms of language and in terms of the research questions and their relevance to the literature before I could recommend

**********

6. PLOS authors have the option to publish the peer review history of their article (what does this mean?). If published, this will include your full peer review and any attached files.

If you choose “no”, your identity will remain anonymous but your review may still be made public.

**Do you want your identity to be public for this peer review?** For information about this choice, including consent withdrawal, please see our Privacy Policy.

Reviewer #1: No

Reviewer #2: No

[NOTE: If reviewer comments were submitted as an attachment file, they will be attached to this email and accessible via the submission site. Please log into your account, locate the manuscript record, and check for the action link "View Attachments". If this link does not appear, there are no attachment files.]

While revising your submission, please upload your figure files to the Preflight Analysis and Conversion Engine (PACE) digital diagnostic tool, https://pacev2.apexcovantage.com/. PACE helps ensure that figures meet PLOS requirements. To use PACE, you must first register as a user. Registration is free. Then, login and navigate to the UPLOAD tab, where you will find detailed instructions on how to use the tool. If you encounter any issues or have any questions when using PACE, please email PLOS at figures@plos.org. Please note that Supporting Information files do not need this step.

# Author comment: Author response to Decision Letter 0

**DOI:** 10.1371/journal.pone.0270503.r002

5 Apr 2022

Dear editor and reviewers,

thank you for taking the time to consider the manuscript and for the valuable feedback. We believe that we have addressed all of your issues now.

Attachment Submitted filename: Response to Reviewers.docx Click here for additional data file.

# Peer review: Decision Letter 1

**DOI:** 10.1371/journal.pone.0270503.r003

**Authors:** Darrell A. Worthy

10 May 2022

PONE-D-21-37441R1Doing Well-Being: 

Self-Reported Activities Are Related to Subjective Well-BeingPLOS ONE

Dear Dr. Nilsson,

Thank you for submitting your manuscript to PLOS ONE. After careful consideration, we feel that it has merit but does not fully meet PLOS ONE’s publication criteria as it currently stands. Therefore, we invite you to submit a revised version of the manuscript that addresses the points raised during the review process.

I received a positive review of your revision from one of the original reviewers.  However, the reviewer lists a few suggestions that should be addressed.  PLOS One does not do extensive copy-editing so please carefully proof-read your manuscript, and attend to the points made by the reviewer.  If you feel that you can address the remaining points made by the reviewer then I invite you to submit a revised manuscript.  

Please submit your revised manuscript by Jun 24 2022 11:59PM. If you will need more time than this to complete your revisions, please reply to this message or contact the journal office at plosone@plos.org. When you're ready to submit your revision, log on to https://www.editorialmanager.com/pone/ and select the 'Submissions Needing Revision' folder to locate your manuscript file.

Please include the following items when submitting your revised manuscript:

- A rebuttal letter that responds to each point raised by the academic editor and reviewer(s). You should upload this letter as a separate file labeled 'Response to Reviewers'.
- A marked-up copy of your manuscript that highlights changes made to the original version. You should upload this as a separate file labeled 'Revised Manuscript with Track Changes'.
- An unmarked version of your revised paper without tracked changes. You should upload this as a separate file labeled 'Manuscript'.

If you would like to make changes to your financial disclosure, please include your updated statement in your cover letter. Guidelines for resubmitting your figure files are available below the reviewer comments at the end of this letter.

If applicable, we recommend that you deposit your laboratory protocols in protocols.io to enhance the reproducibility of your results. Protocols.io assigns your protocol its own identifier (DOI) so that it can be cited independently in the future. For instructions see: https://journals.plos.org/plosone/s/submission-guidelines#loc-laboratory-protocols. Additionally, PLOS ONE offers an option for publishing peer-reviewed Lab Protocol articles, which describe protocols hosted on protocols.io. Read more information on sharing protocols at https://plos.org/protocols?utm_medium=editorial-email&utm_source=authorletters&utm_campaign=protocols.

We look forward to receiving your revised manuscript.

Kind regards,

Darrell A. Worthy, Ph.D

Academic Editor

PLOS ONE

Journal Requirements:

Please review your reference list to ensure that it is complete and correct. If you have cited papers that have been retracted, please include the rationale for doing so in the manuscript text, or remove these references and replace them with relevant current references. Any changes to the reference list should be mentioned in the rebuttal letter that accompanies your revised manuscript. If you need to cite a retracted article, indicate the article’s retracted status in the References list and also include a citation and full reference for the retraction notice.

[Note: HTML markup is below. Please do not edit.]

Reviewers' comments:

Reviewer's Responses to Questions

**Comments to the Author**

1. If the authors have adequately addressed your comments raised in a previous round of review and you feel that this manuscript is now acceptable for publication, you may indicate that here to bypass the “Comments to the Author” section, enter your conflict of interest statement in the “Confidential to Editor” section, and submit your "Accept" recommendation.

Reviewer #1: All comments have been addressed

**********

2. Is the manuscript technically sound, and do the data support the conclusions?

The manuscript must describe a technically sound piece of scientific research with data that supports the conclusions. Experiments must have been conducted rigorously, with appropriate controls, replication, and sample sizes. The conclusions must be drawn appropriately based on the data presented. 

Reviewer #1: Yes

**********

3. Has the statistical analysis been performed appropriately and rigorously? 

Reviewer #1: Yes

**********

4. Have the authors made all data underlying the findings in their manuscript fully available?

The PLOS Data policy requires authors to make all data underlying the findings described in their manuscript fully available without restriction, with rare exception (please refer to the Data Availability Statement in the manuscript PDF file). The data should be provided as part of the manuscript or its supporting information, or deposited to a public repository. For example, in addition to summary statistics, the data points behind means, medians and variance measures should be available. If there are restrictions on publicly sharing data—e.g. participant privacy or use of data from a third party—those must be specified.

Reviewer #1: Yes

**********

5. Is the manuscript presented in an intelligible fashion and written in standard English?

PLOS ONE does not copyedit accepted manuscripts, so the language in submitted articles must be clear, correct, and unambiguous. Any typographical or grammatical errors should be corrected at revision, so please note any specific errors here.

Reviewer #1: Yes

**********

6. Review Comments to the Author

Please use the space provided to explain your answers to the questions above. You may also include additional comments for the author, including concerns about dual publication, research ethics, or publication ethics. (Please upload your review as an attachment if it exceeds 20,000 characters)

Reviewer #1: Review of PONE-D-21-37441R1

I was a reviewer (1) of the previous paper, and I thank the authors for their considered responses and changes to all my previous remarks. I think the paper is now more accessible and, given that it was always interesting and cleverly done, I have no suggestions for further major change. The paper will remain a little difficult for many readers but this reflects the innovative and unfamiliar work undertaken.

I thought the English was always understandable. I do suggest below a patch for some of the abstract, which might improve this a little. It’s below and should be OK to cut & paste if the authors choose to.

Abstract patch:

In Study 2 (N=295) individuals showed strong agreement concerning which activities they considered to have increased or decreased SWB (AUC = .995). Words describing activities that increased SWB related to physically and cognitively active activities and social activities (“football”, “meditation”, “friends”), whereas words describing activities that decreased SWB mainly illustrated activity features related to imbalance (“too”, “much”, “enough”).

On the reread I spent a bit of time thinking about eating (& various related words) in Figures 1 and 2. These activities don’t seem to do much for SWB but SWB probably would decrease it if you didn’t do them. Everyone (well, nearly everyone) eats daily and most people are reasonably happy so why would you notice a connection? Why would there be one when there is little variance? On a somewhat different track I wondered if the eating results in Figure 2 would be a bit more positive if you ran the study somewhere other than the UK. At least anecdotally most other cultures do regard food as a source of SWB. I don’t think you should feel obliged to include these points in this paper, but they might be worth considering in the future.

Nice paper!

**********

7. PLOS authors have the option to publish the peer review history of their article (what does this mean?). If published, this will include your full peer review and any attached files.

If you choose “no”, your identity will remain anonymous but your review may still be made public.

**Do you want your identity to be public for this peer review?** For information about this choice, including consent withdrawal, please see our Privacy Policy.

Reviewer #1: No

[NOTE: If reviewer comments were submitted as an attachment file, they will be attached to this email and accessible via the submission site. Please log into your account, locate the manuscript record, and check for the action link "View Attachments". If this link does not appear, there are no attachment files.]

While revising your submission, please upload your figure files to the Preflight Analysis and Conversion Engine (PACE) digital diagnostic tool, https://pacev2.apexcovantage.com/. PACE helps ensure that figures meet PLOS requirements. To use PACE, you must first register as a user. Registration is free. Then, login and navigate to the UPLOAD tab, where you will find detailed instructions on how to use the tool. If you encounter any issues or have any questions when using PACE, please email PLOS at figures@plos.org. Please note that Supporting Information files do not need this step.

# Author comment: Author response to Decision Letter 1

**DOI:** 10.1371/journal.pone.0270503.r004

21 May 2022

Thank you for reviewing this manuscript once again. We appreciate the positive feedback, suggestions for improvement and the interesting reflections.

Attachment Submitted filename: Response to Reviewers - revision 2 .docx Click here for additional data file.

# Editor report: Decision Letter 2

**DOI:** 10.1371/journal.pone.0270503.r005

**Authors:** Darrell A. Worthy

13 Jun 2022

Doing Well-Being: 

Self-Reported Activities Are Related to Subjective Well-Being

PONE-D-21-37441R2

Dear Dr. Nilsson,

We’re pleased to inform you that your manuscript has been judged scientifically suitable for publication and will be formally accepted for publication once it meets all outstanding technical requirements.

Within one week, you’ll receive an e-mail detailing the required amendments. When these have been addressed, you’ll receive a formal acceptance letter and your manuscript will be scheduled for publication.

An invoice for payment will follow shortly after the formal acceptance. To ensure an efficient process, please log into Editorial Manager at http://www.editorialmanager.com/pone/, click the 'Update My Information' link at the top of the page, and double check that your user information is up-to-date. If you have any billing related questions, please contact our Author Billing department directly at authorbilling@plos.org.

If your institution or institutions have a press office, please notify them about your upcoming paper to help maximize its impact. If they’ll be preparing press materials, please inform our press team as soon as possible -- no later than 48 hours after receiving the formal acceptance. Your manuscript will remain under strict press embargo until 2 pm Eastern Time on the date of publication. For more information, please contact onepress@plos.org.

Kind regards,

Darrell A. Worthy, Ph.D

Academic Editor

PLOS ONE

Additional Editor Comments (optional):

Reviewers' comments:

# Editor report: Acceptance letter

**DOI:** 10.1371/journal.pone.0270503.r006

**Authors:** Darrell A. Worthy

15 Jun 2022

PONE-D-21-37441R2 

Doing Well-Being:Self-Reported Activities Are Related to Subjective Well-Being 

Dear Dr. Nilsson:

I'm pleased to inform you that your manuscript has been deemed suitable for publication in PLOS ONE. Congratulations! Your manuscript is now with our production department. 

If your institution or institutions have a press office, please let them know about your upcoming paper now to help maximize its impact. If they'll be preparing press materials, please inform our press team within the next 48 hours. Your manuscript will remain under strict press embargo until 2 pm Eastern Time on the date of publication. For more information please contact onepress@plos.org.

If we can help with anything else, please email us at plosone@plos.org. 

Thank you for submitting your work to PLOS ONE and supporting open access. 

Kind regards, 

PLOS ONE Editorial Office Staff

on behalf of

Dr. Darrell A. Worthy 

Academic Editor

PLOS ONE
